# Supplementary material for: Design of efficacious somatic cell genome editing strategies for recessive and polygenic diseases
Source: Nat Commun. 2020 Dec 8;11:6277. doi: 10.1038/s41467-020-20065-8 (PMC7722885; doi:10.1038/s41467-020-20065-8)
Supplement: Supplementary file 1 — Supplementary Information [file 41467_2020_20065_MOESM1_ESM.pdf]

Design of efficacious somatic cell genome editing strategies for recessive and polygenic diseases

Jared Carlson-Stevermer[1,2*], Amritava Das[1,3*], Amr A. Abdeen[1], David Fiflis[1,2], Benjamin I Grindel[1,2], Shivani Saxena[1,2], Tugce Akcan[4], Tausif Alam[4], Heidi Kletzien[2], Lucille Kohlenberg[1], Madelyn Goedland[1,2], Micah J. Dombroe[1], Krishanu Saha[1,2,5]

[1] Wisconsin Institute for Discovery, University of Wisconsin-Madison, Madison, WI, USA

[2] Department of Biomedical Engineering, University of Wisconsin-Madison, Madison, WI, USA

[3] Morgridge Institute for Research, Madison, WI, USA

[4] Department of Surgery, University of Wisconsin, Madison WI, USA

[5] Retina Research Foundation Kathryn and Latimer Murfee Chair, Madison WI, USA

* indicates equal contribution

Contents

Supplementary Figures 1-15

Supplementary Notes

Supplementary Video File Legends

Supplementary Tables 1-10

Link to Zenodo online files: https://tinyurl.com/GETEMZenodo (contains Supplementary Videos, Modeling Code, Sequencing Data, and Western Blot image)

Supplementary Figures

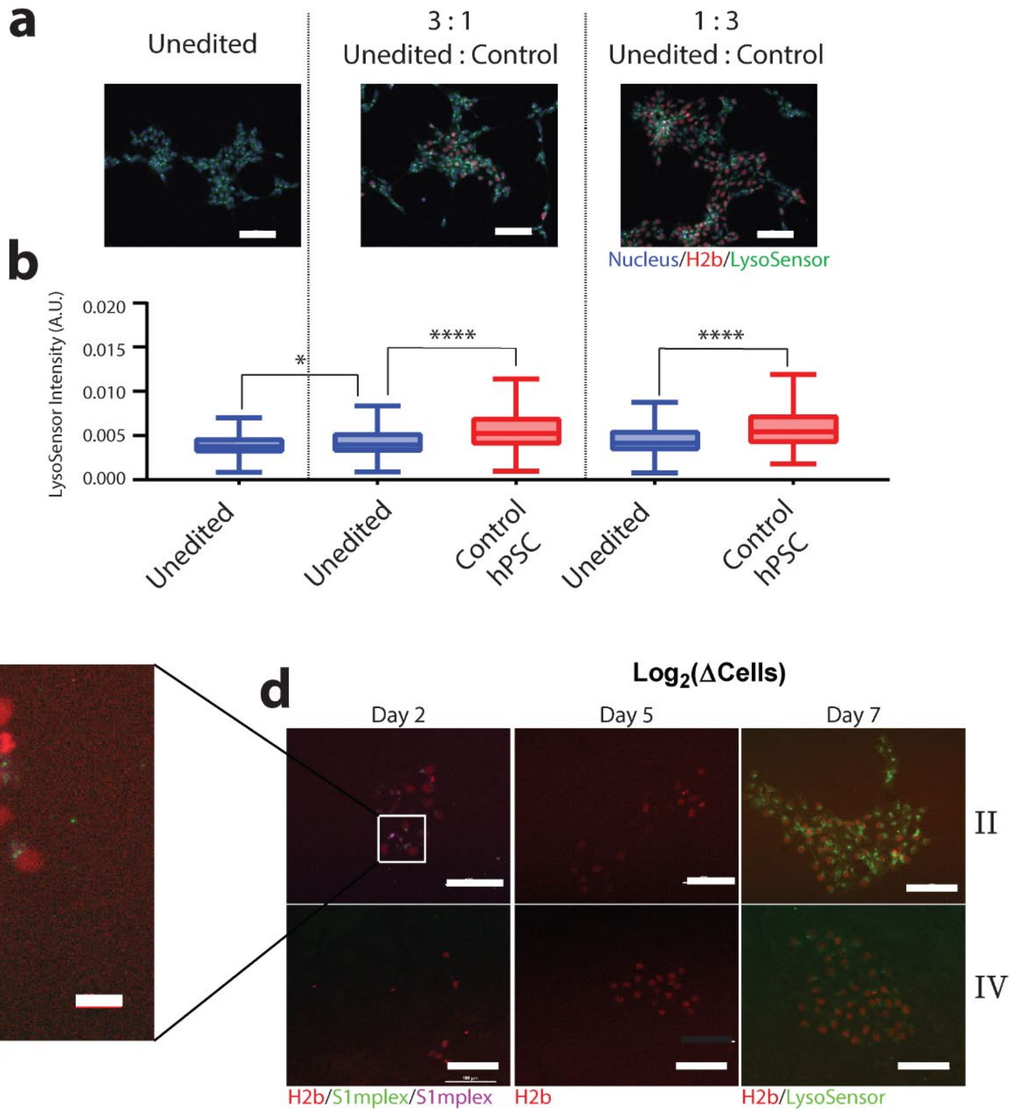

Supplementary Figure 1 | Use of ArrayEdit for isolation of the gene-corrected iPSCs. a, Representative images of hPSCs following LysoSensor Green staining. Unedited cells were either cultured alone, or at the indicated ratio with normal hPSC-H2B labelled cells (Control) to identify each cell line (*scale bar: 100 µm*). **b,** Quantification of LysoSensor intensity in each co-culture condition. LysoSensor intensity was measured on a per-cell basis using confocal microscopy. Control hPSCs had significantly higher intensity than unedited cells in all conditions. Unedited cells co-cultured with hPSCs also had an increased LysoSensor intensity when compared to those cultured alone (two-tailed test, *p<0.05, ****p<0.001). **c,** Representative µFeatures from ArrayEdit representing populations from quadrants 2 and 4 from panel c. S1mplexes can be seen in the nucleus as late as day 2 and form the basis on which feature to select (*scale bar: 100µm*). **d,** Magnification of Day 2 colony. S1mplexes can be seen throughout the image (*scale bar: 25 µm)*.

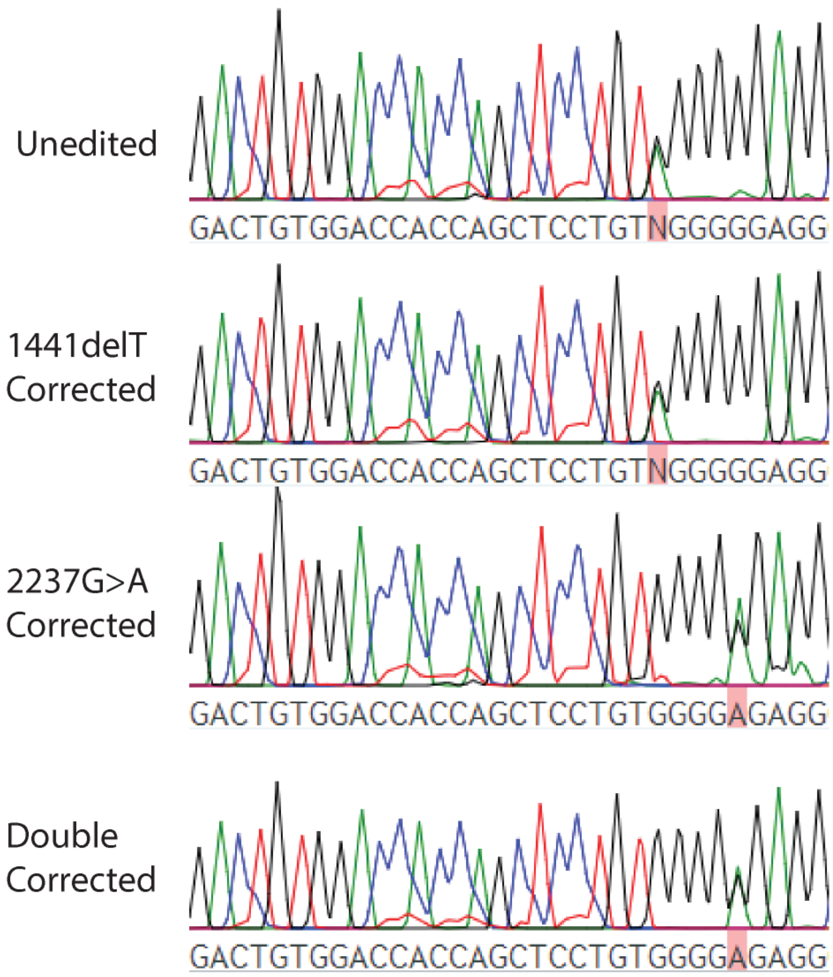

Supplementary Figure 2 | Characterization of the gene-corrected lines: Long PCR sequence traces show SNPs indicating that both alleles were magnified. Sanger sequencing traces of long range PCR shown in Fig. 2. SNPs were observed showing that PCR products were a result of amplification from both alleles within the cells.

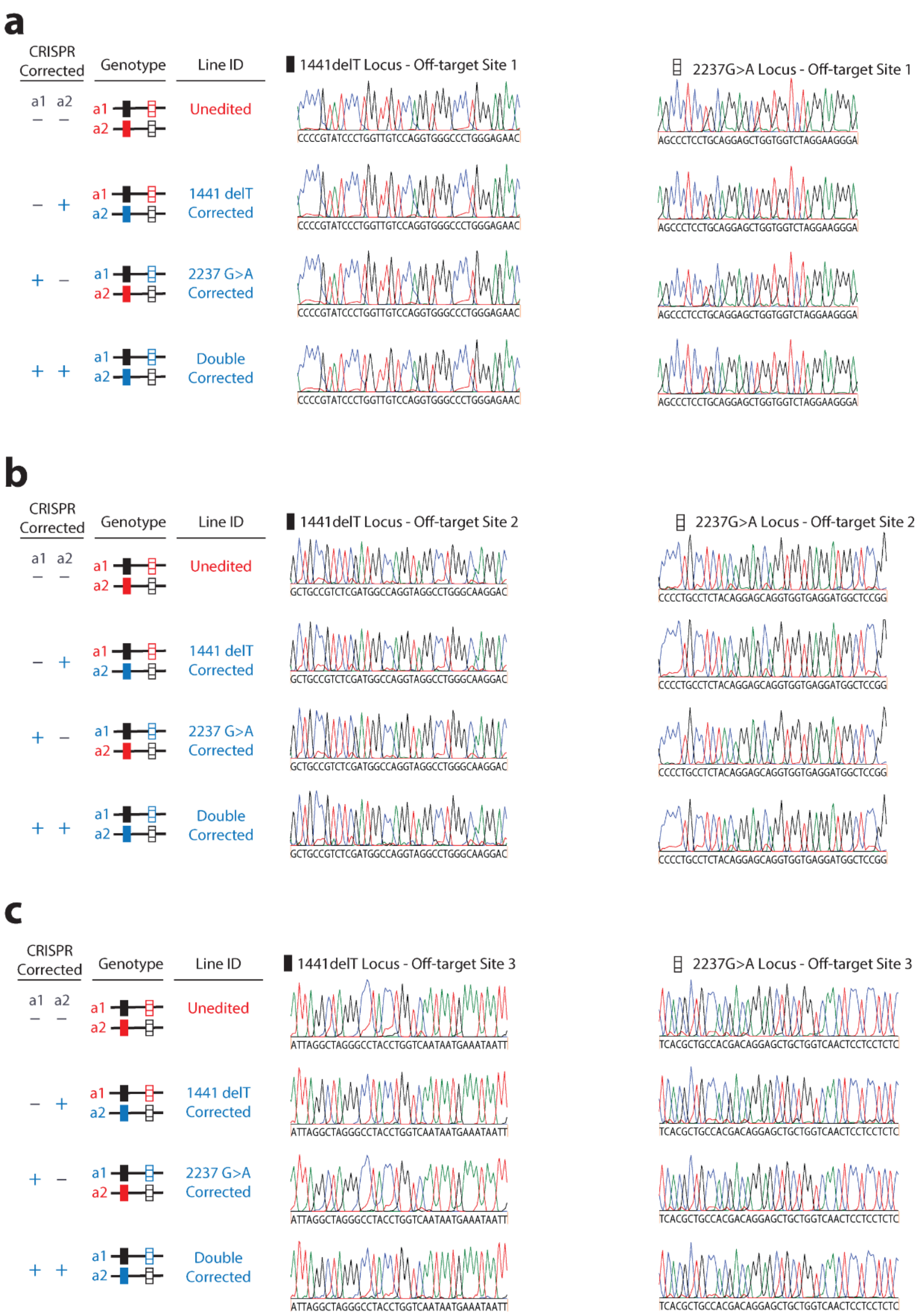

Supplementary Figure 3 | Off-target analysis of the gene-corrected lines. Off-target analysis of top 3 off-target sites for both sgRNAs. Sanger sequencing was unable to detect any deviations at off-target sites in any of the corrected lines in comparison to unedited cells.

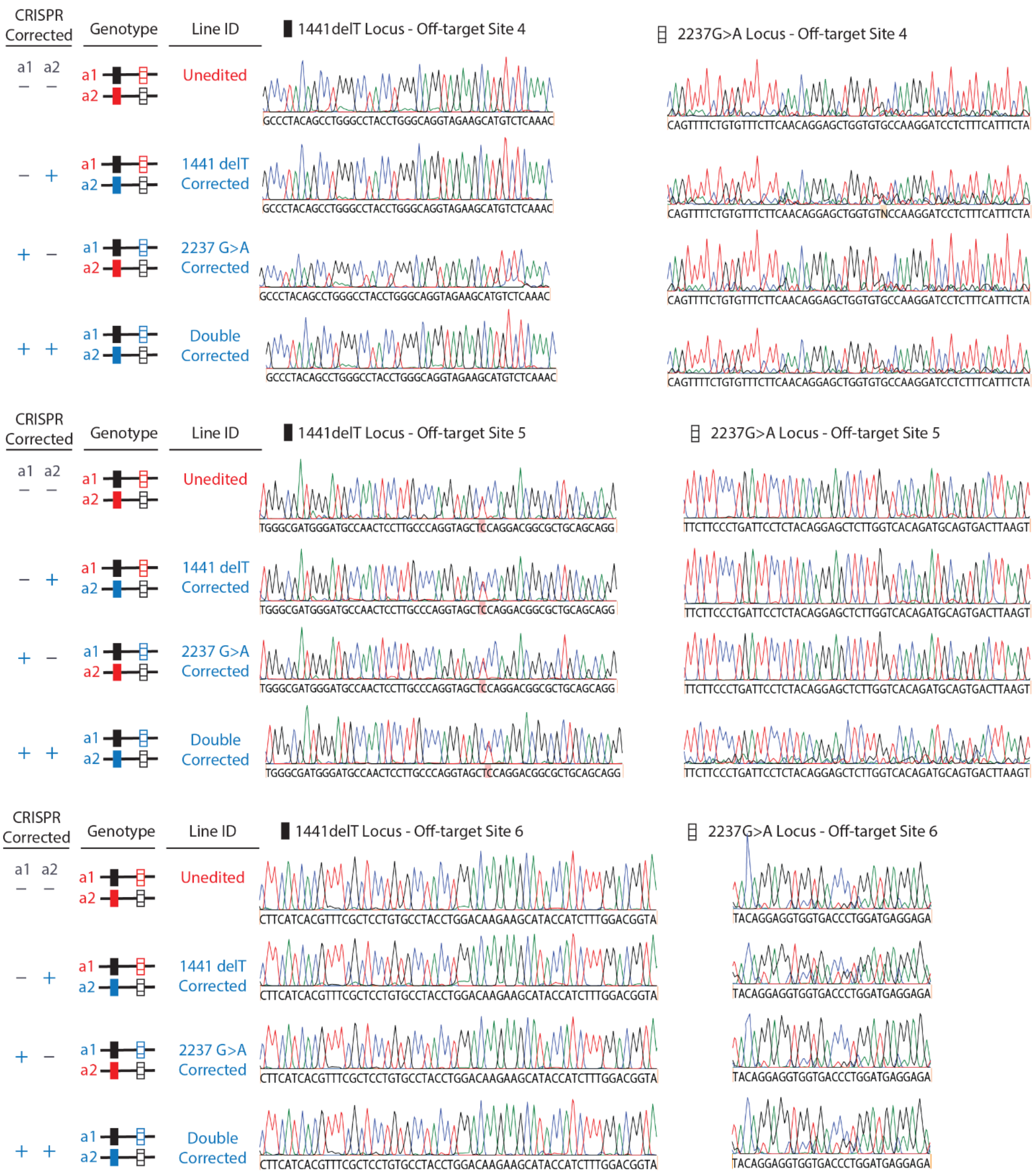

Supplementary Figure 4 | Off-target analysis of the gene-corrected lines. Off-target analysis of top 4th, 5th and 6th off-target sites for both sgRNAs. Sanger sequencing was unable to detect any deviations at off-target sites in any of the corrected lines in comparison to unedited cells.

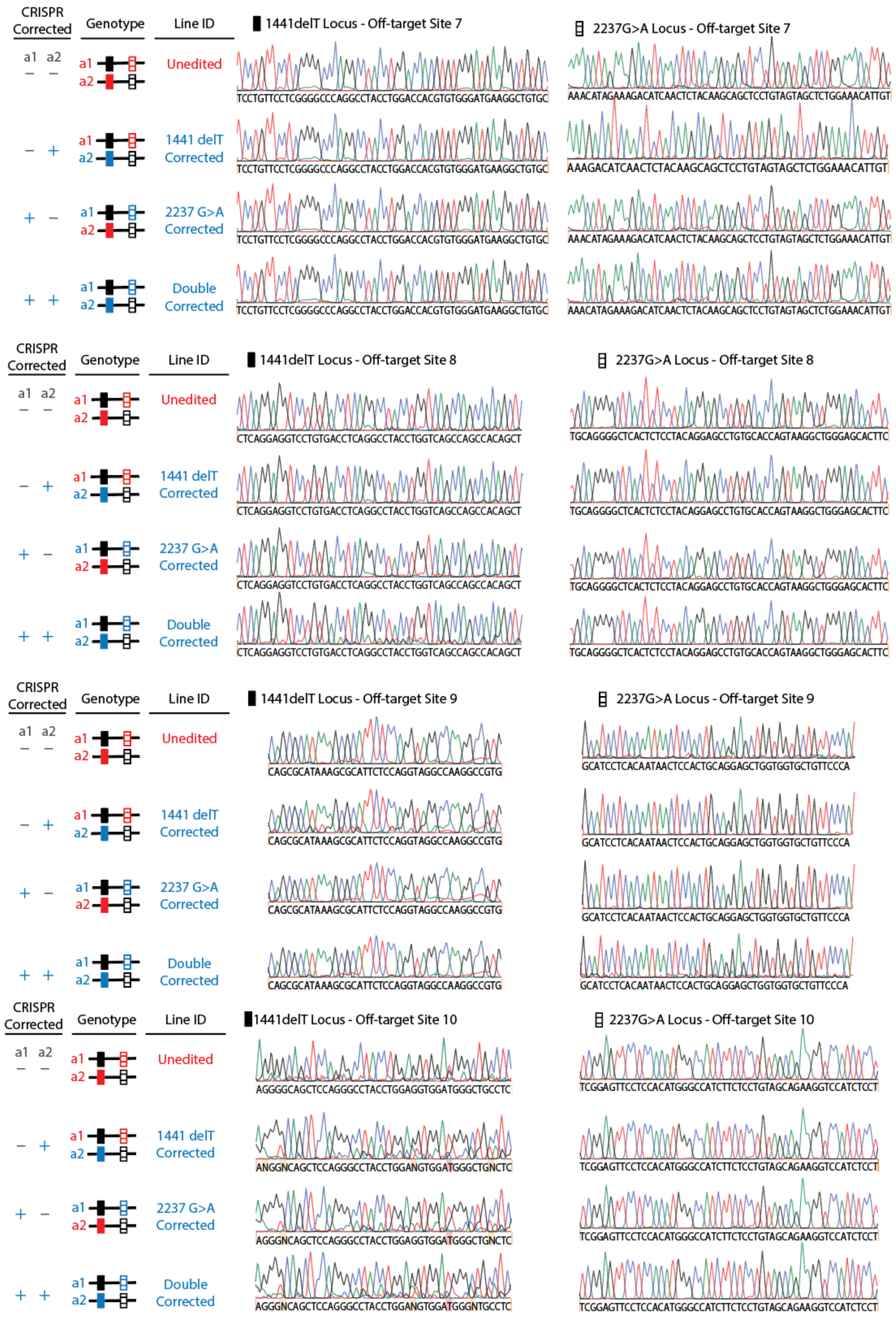

Supplementary Figure 5 | Off-target analysis of the gene-corrected lines. Off-target analysis of top 7th, 8th, 9th and 10th off-target sites for both sgRNAs. Sanger sequencing was unable to detect any deviations at off-target sites in any of the corrected lines in comparison to unedited cells.

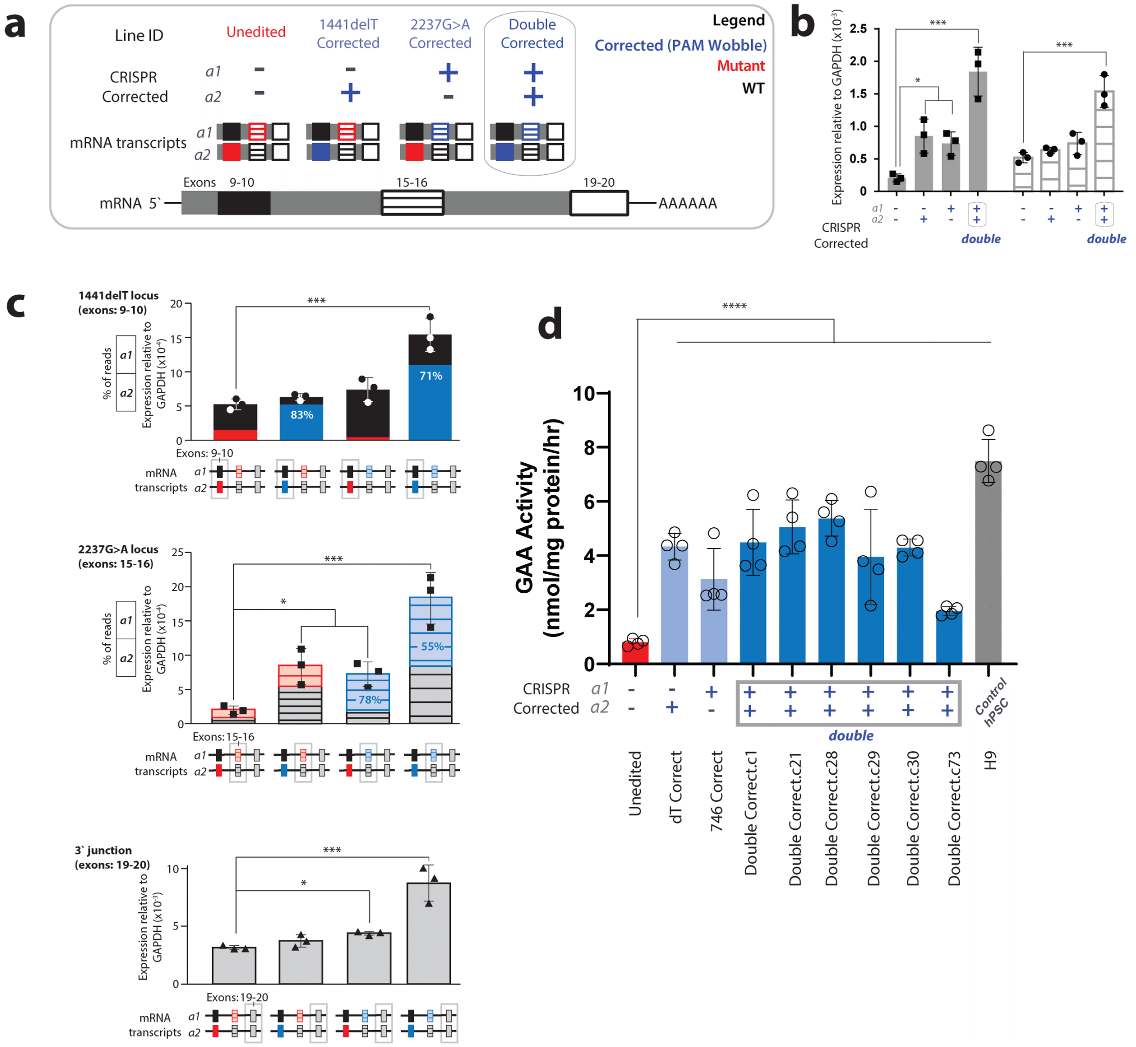

Supplementary Figure 6 | Gene correction increases mRNA expression from corrected allele and secreted GAA. a, Schematic indicating the genotypes of the iPSC lines generated and mRNA transcripts collected. **b,** qRT-PCR analysis around both mutated alleles. Expression was increased in single corrected lines at the 1447delT locus (solid) by 4-fold and in double corrections by 10-fold over unedited cells. In comparison, expression at the 2237G>A locus (hashed) was not significantly different between unedited and single corrected lines. Double corrected lines saw a 3-fold increase in expression (*p<0.05, ***p<0.005, n=3 technical replicates). **c,** *Top panel:* Schematic of *GAA* mRNA used for qRT-PCR. mRNA was analyzed at 3 locations, around the 1441delT locus (solid), the 2237G>A locus (hashed), and at the final 3' junction (outlined). *Middle two panels:* Overlay of qRT-PCR and deep sequencing data around each edited locus: analysis around 1441delT loci (solid bars) and analysis around 2237G>A locus (hashed bars). Bars are color coded by sequence identity, either wildtype, mutant, or corrected, from deep sequencing analysis. Bar heights are equivalent to qRT-PCR quantification relative to *GAPDH.* In all corrected lines, the corrected allele was expressed at a higher frequency than the unedited allele. Double corrected line expressed the highest level of overall mRNA and expressed each allele at approximately equal amounts. *Bottom panel:* Quantification of total *GAA* mRNA in unedited, single corrected, and double corrected lines via qRT-PCR. The double corrected line had a significantly higher amount of mRNA than any of the other isolated line (n=3 technical replicates). This is consistent with expression from two active alleles (*p<0.05 ***p<0.005, two-tailed t-test, α = 0.05, heteroscedastic, mean ± s.d). **d,** GAA activity in cell culture media supernatant as measured by 4-MUG cleavage in acidic conditions. All corrected lines had significantly higher activity than unedited cells but were indistinguishable from each other (n=5 technical replicates, ****p<0.0001, two-tailed t-test, α = 0.05, heteroscedastic; , mean ± s.d).

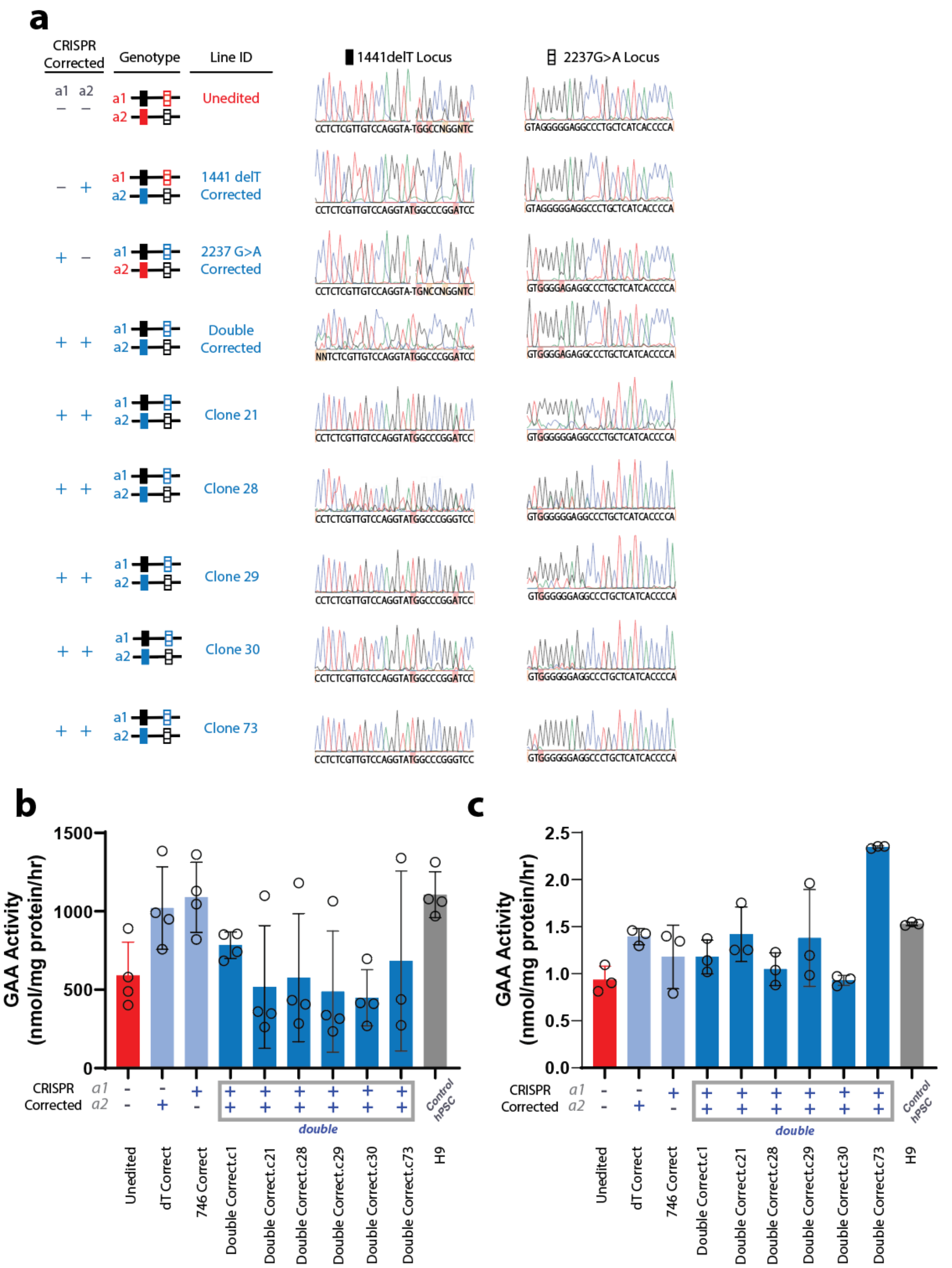

Supplementary Figure 7 | Multiple double corrected clones generated using several methods. a, Sanger sequencing traces at both loci for clones generated using ArrayEdit, and other methods. Corrected cell lines include Double Correct.c1 (isolated via ArrayEdit), Double Correct.c21, Double Correct.c28, Double Correct.c29, Double Correct.c30 (isolated via sequential correction of 2237G>A allele followed by s1mplex electroporation correction of 1440delT) and Double Correct.c73(isolated via sequential correction of 2237G>A allele followed by using transient puromycin based correction of 1440delT). **b,** GAA activity in cell lysate as measured by 4-MUG cleavage in neutral conditions (n=4 technical replicates; , mean ± s.d). **c,** GAA activity in cell culture media supernatant as measured by 4-MUG cleavage in neutral conditions. (n=3 technical replicates; , mean ± s.d).

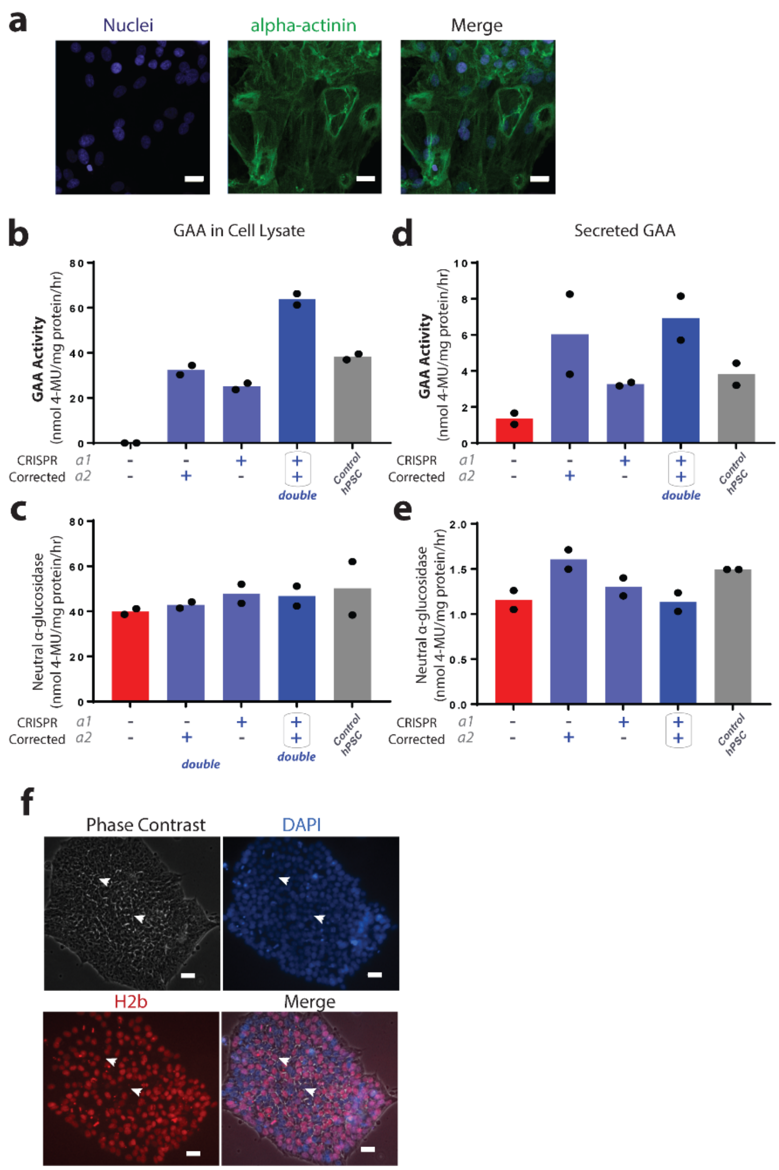

Supplementary Figure 8 | Phenotypic recovery of GAA activity in differentiated cardiomyocytes. a, Immunocytochemistry for sarcomeric α-actinin in differentiated unedited iPSC-CMs. Striation pattern indicative of cardiomyocyte differentiation can be observed (*scale bar: 25µm*). **b,** GAA activity in cell lysate of cardiomyocytes as measured by 4-MUG cleavage in acidic conditions. Unedited cells were unable to cleave this substrate, showing there was little to no active protein. All corrected lines were indistinguishable from each other (n=2 technical replicates). **c,** GAA activity in cell lysate as measured by 4-MUG cleavage in neutral conditions. All lines had equal activity compared to unedited cells (n=2 technical replicates). **d,** GAA activity in spent media as measured by 4-MUG cleavage in acidic conditions. Unedited cells were unable to cleave this substrate, showing there was little to no active secreted protein. (n=2 technical replicates). **e,** GAA activity in spent media as measured by 4-MUG cleavage in neutral conditions. All lines had equal activity compared to unedited cells (n=2 technical replicates). **f,** Silencing of active transgenes at *AAVS1* locus. A CAGGS-H2B-mCherry reporter was integrated into the safe-harbor locus *AAVS1* promoting constitutive expression of this fluorophore. After 30 passages, this expression was silenced in a significant number of hPSCs (arrowheads) (*scale bar: 25µm*).

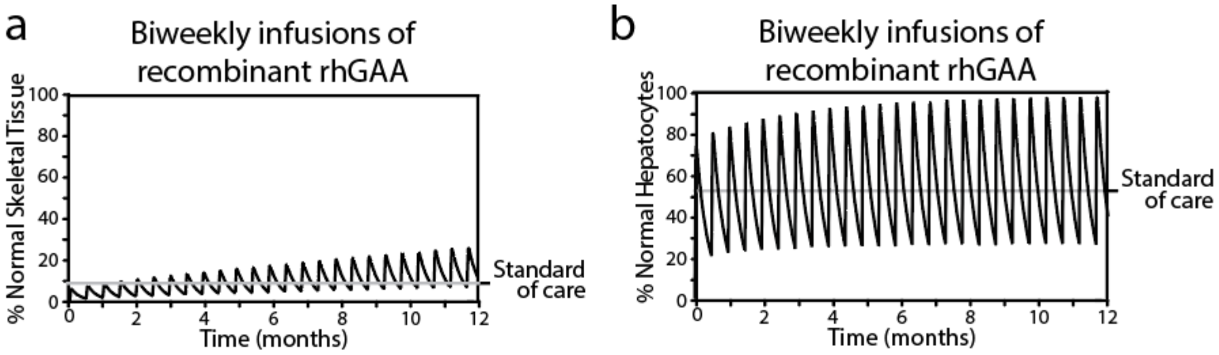

Supplementary Figure 9 | *In silico* simulation of Enzyme Replacement Therapy. a, Percentage of normal skeletal tissue within a heart of a Pompe diseased infant after 1 year of biweekly ERT at 20 mg/kg. **b,** Percentage of normal hepatocytes within a heart of a Pompe diseased infant after 1 year of biweekly ERT at 20 mg/kg.

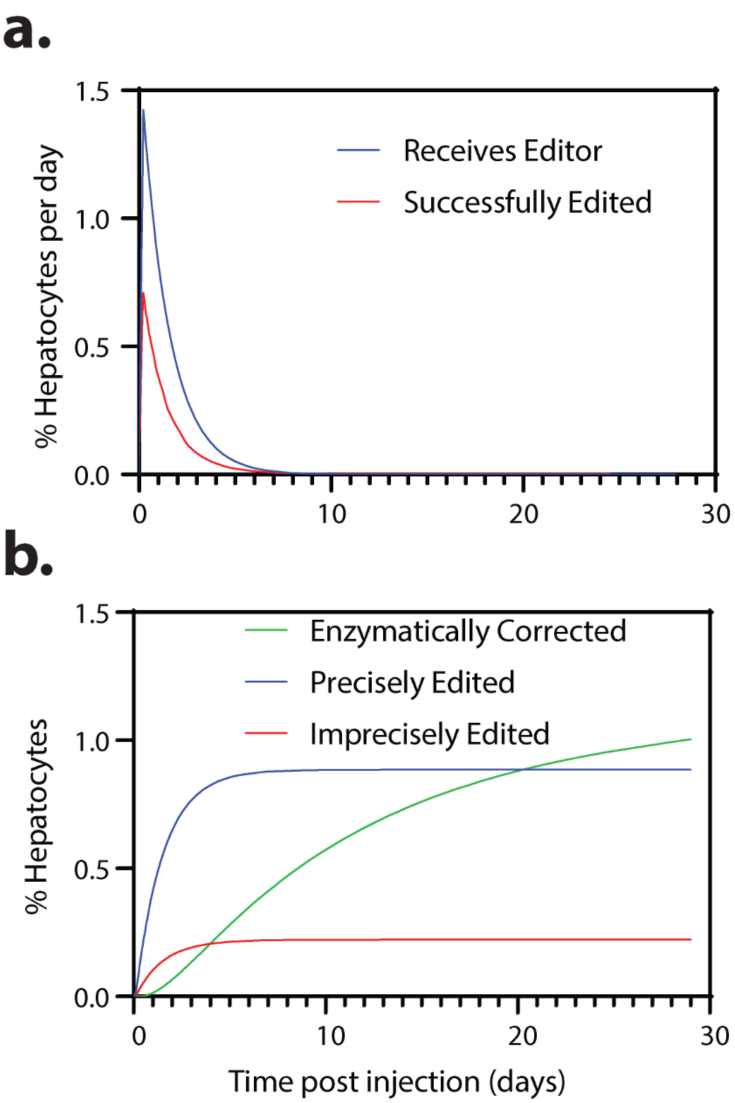

Supplementary Figure 10 | GETEM for Pompe disease correction demonstrates the differences in timescales in genome editing driven rescue and cross-correction driven rescue. a, Rate of liver genome editing expressed as the percentage of the hepatocytes interacting with the genome editor per day along with the change of the percentage of edited (precise and imprecise) hepatocytes per day. This indicates that under 1.5 % of the liver interacts with the genome editor, and that the reach of the genome editor is limited. The total percentage of cells reached by the genome editor is about 0.8% by integrating this curve. **b,** The percentage of the liver that is precisely edited, imprecisely edited and enzymatically cross-corrected after a single dose of one genome editor. While the percentage of genome edited cells stabilize within 5 days, the enzymatic cross correction continues to rise monotonically due to secreted GAA buildup and absorption. While the percentage of edited cells stabilizes within a few days, the percentage of enzymatically cross-corrected cells continues to rise monotonically throughout the month analyzed.

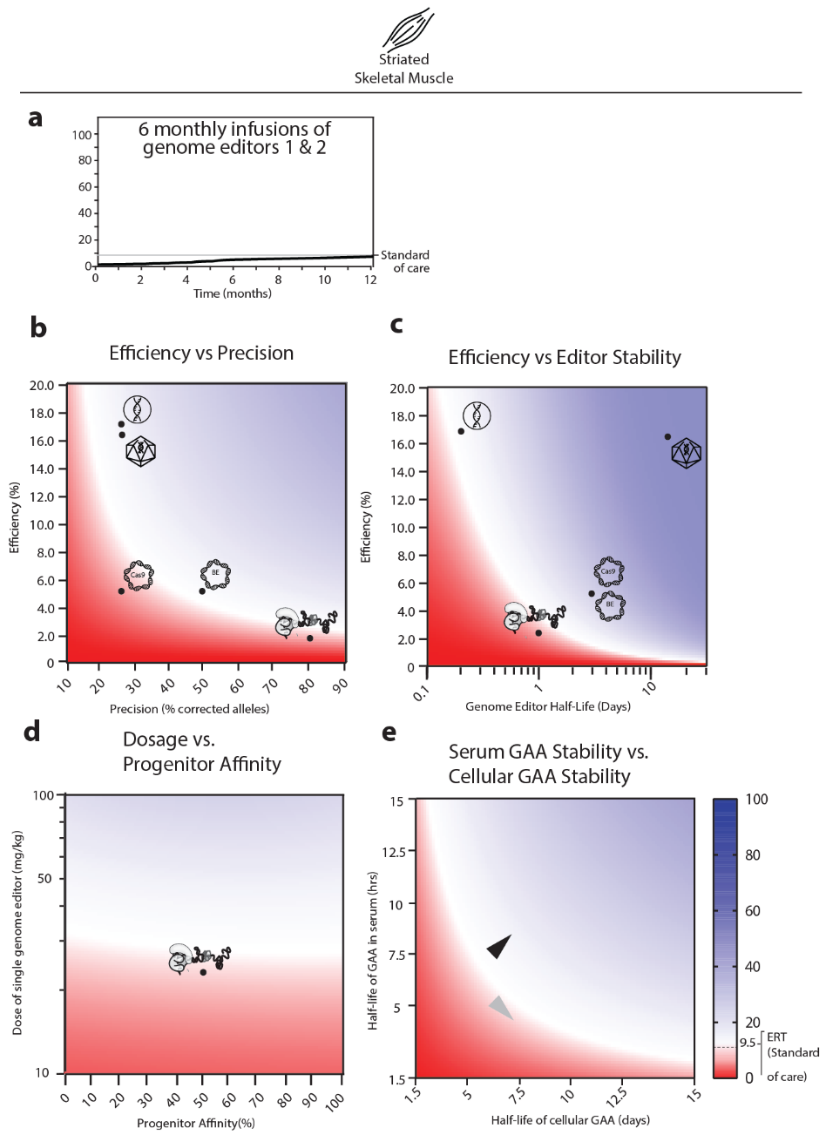

Supplementary Figure 11 | GETEM for Pompe disease correction in the striated skeletal muscle. a, Percentage of normal tissue within the developing striated skeletal muscle tissue of a Pompe diseased infant after the administration of six doses of genome editors at 107 mg/kg. **b**, Trade-off between genome editor efficiency and precision focusing on the percentage of corrected muscle tissue. Heatmap indicates that lower efficiencies can be efficacious if higher precision editors are used. **c**, Trade-off between editor genome efficiency and genome editor stability, focusing on the percentage of corrected skeletal muscle tissue. Heatmap indicates that lower efficiency editors could be efficacious if the extracellular editor stability increases. **d,** Trade-off between increasing genome editor dose and progenitor affinity focusing on the percentage of corrected muscle tissue. Heatmap indicates that lower doses can be used with delivery strategies preferentially targeting progenitors. **e,** Using GETEM, heatmap indicating trade-off in skeletal muscle correction in the developing infant between the degradation rates of GAA in the serum and cellular GAA, indicating that stabilization of GAA in the serum to increase its half-life by even 2 hours can improve clinical outcome. (grey arrowhead indicates pre-stabilization, black arrowhead indicates post-stabilization). Heatmap scale based on 20 mg/kg rhGAA ERT, which corrects approximately 9.6% of skeletal muscle tissue (estimated, see **Supplementary Notes, Supplementary Figure 9**).

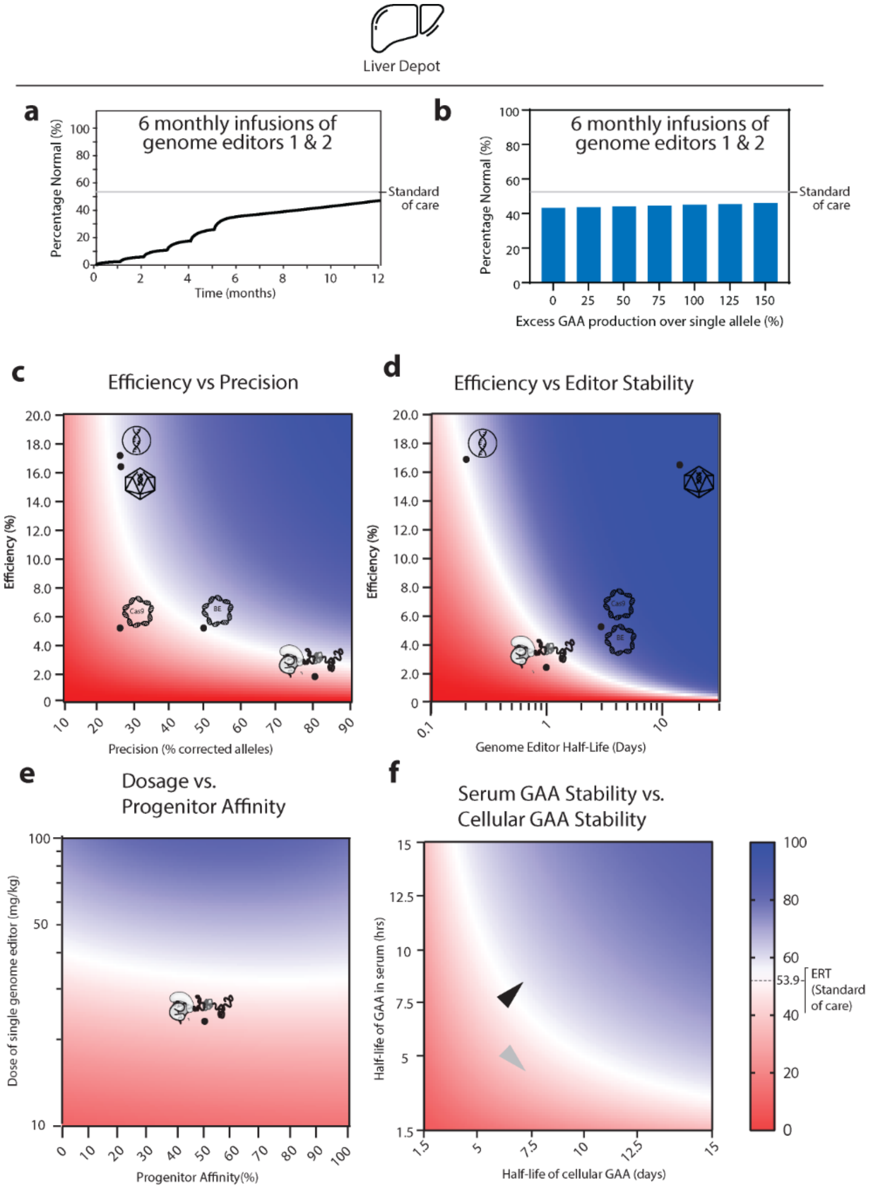

Supplementary Figure 12 | GETEM for Pompe disease correction in the liver depot. a, Percentage of normal hepatocytes within the developing liver of a Pompe diseased infant after the administration of six doses of genome editors at 107 mg/kg. **b**, Bar chart showing the increase in the normal hepatocytes in situations ranging from double corrected cells produce no more GAA than single corrected cells to double corrected cells producing 150% more GAA than single corrected cells. Since double corrected cells form about 1% of the hepatocytes, their effect on the amelioration of the disease phenotype is minimal **c**, Trade-off between genome editor efficiency and precision focusing on the percentage of normal hepatocytes. Heatmap indicates that lower efficiencies can be efficacious if higher precision editors are used. **d**, Trade-off between editor genome efficiency and genome editor stability, focusing on the percentage of normal hepatocytes. Heatmap indicates that lower efficiency editors could be efficacious if the extracellular editor stability increases. **e,** Trade-off between increasing genome editor dose and progenitor affinity focusing on the percentage of normal hepatocytes. Heatmap indicates that lower doses can be used with delivery strategies preferentially targeting progenitors. **f,** Using GETEM, heatmap indicating trade-off in hepatocyte correction in the developing infant between the degradation rates of GAA in the serum and cellular GAA, indicating that stabilization of GAA in the serum to increase its half-life by even 2 hours can improve clinical outcome. (grey arrowhead indicates pre-stabilization, black arrowhead indicates post-stabilization). Heatmap scale based on 20 mg/kg rhGAA ERT, which corrects approximately 53.5% of hepatocytes (estimated, see **Supplementary Notes, Supplementary Figure 9**).

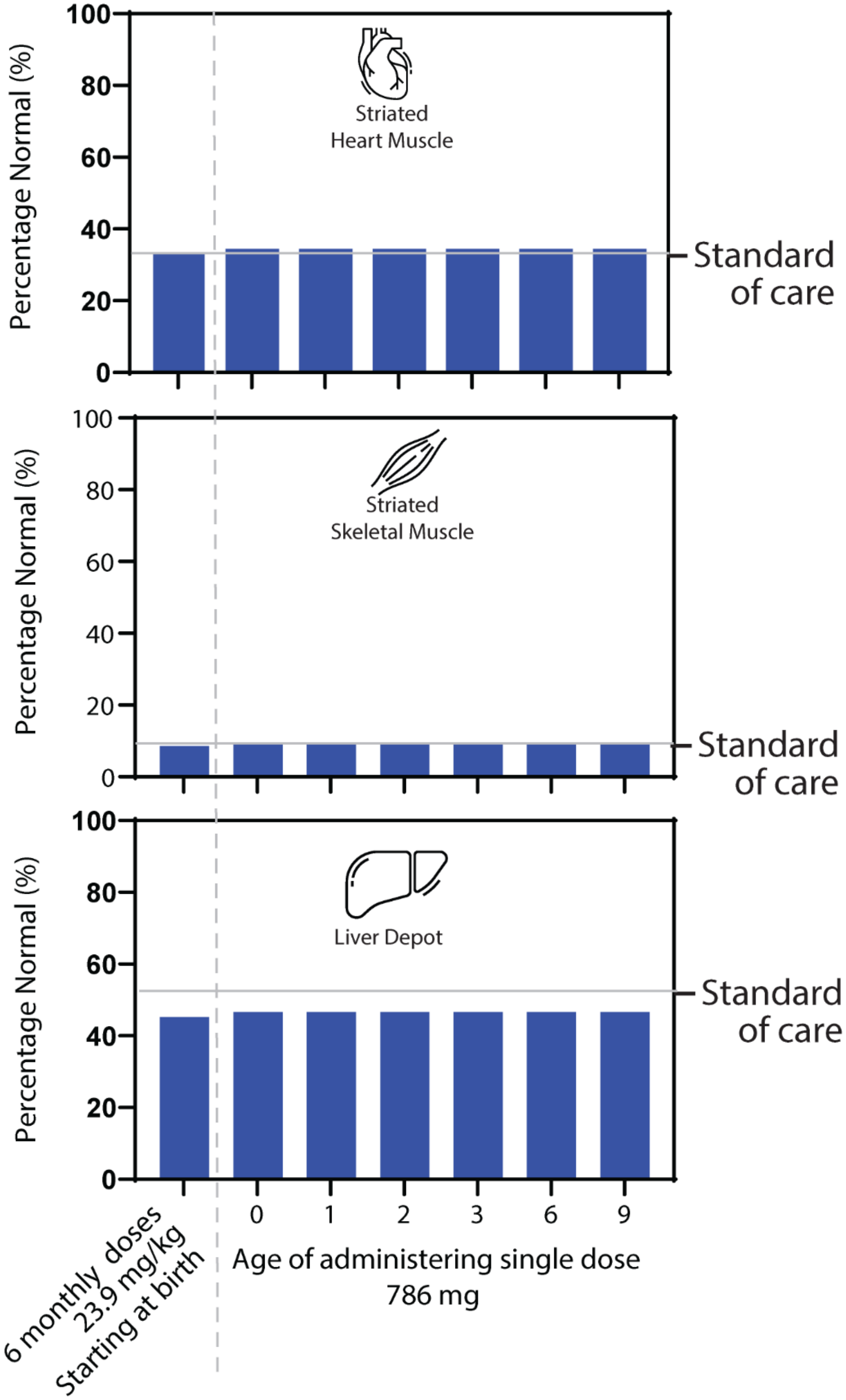

Supplementary Figure 13 | GETEM for Pompe disease correction using single large dose rather than multiple doses. Minimal differences are observed when 6 monthly doses of 107 mg/kg are delivered over 5 months v. delivering the equivalent amount of genome editor in a single bolus at ages 0 – 9 months. Data shown for striated cardiac tissue, striated muscle tissue and the liver depot, along with appropriate standard of care based on 20 mg/kg ERT (**Fig. 5**, and **Supplementary Figure 9**)

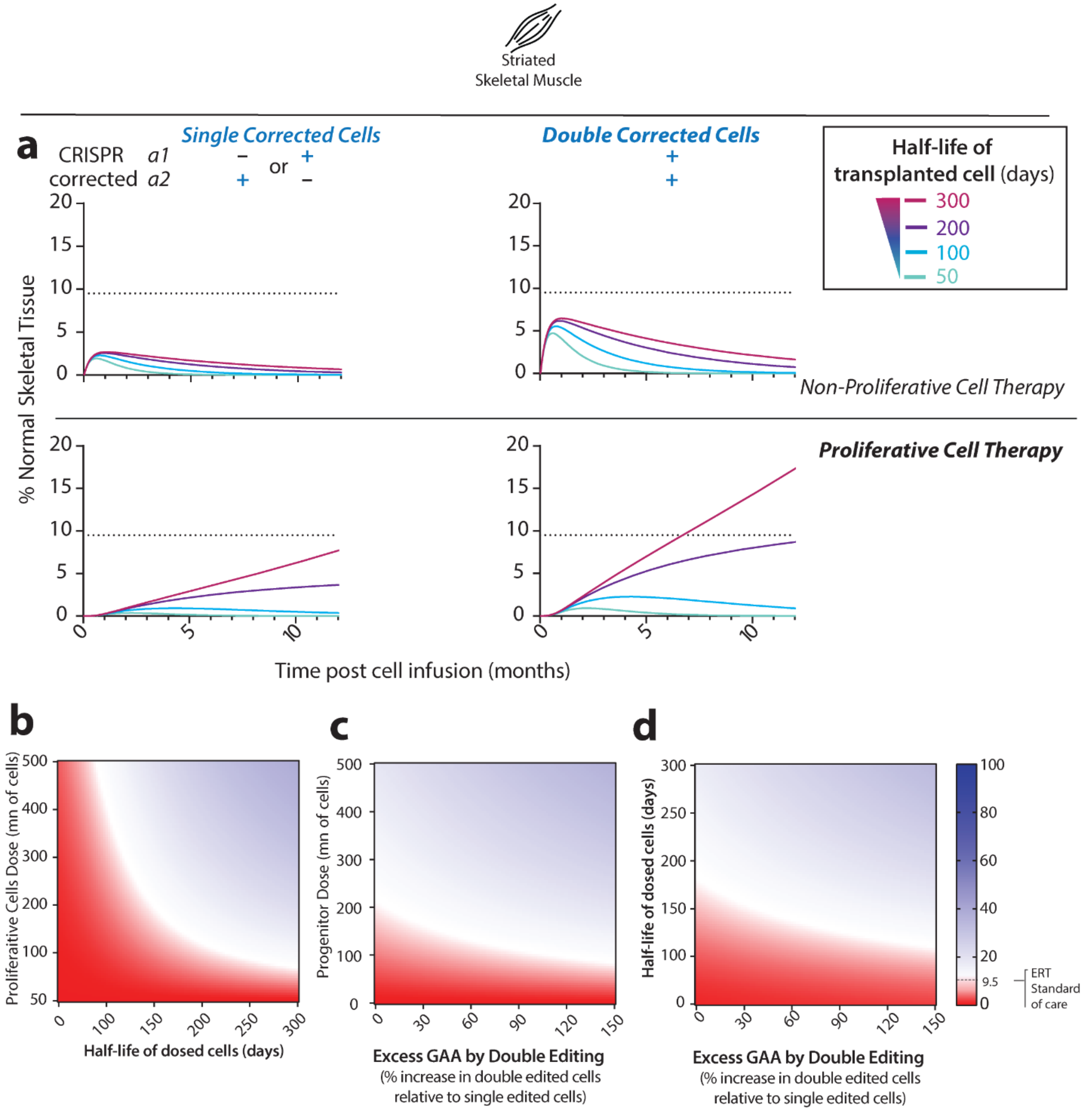

Supplementary Figure 14 | Cell therapy for Pompe disease requires persistent proliferative cells a, Plot showing the degree of phenotypically normal skeletal muscle tissue when either 10 billion non-proliferative single or double corrected cells are dosed or 100 million proliferative single or double corrected cells are dosed, , the double corrected cells whose GAA production is 150% excess relative to single corrected cells, for 4 different half-lives of dosed cells. For long lasting correction, proliferative progenitor cells need to have a half-life exceeding 100 days. **b,** Heatmap showing the proliferative cell dose (assuming double corrected cells have 75% excess GAA production relative to single corrected cells) against the half-life of dosed cells. If the dosed cells have a half-life similar to endogenous hepatocytes, a 75 million cell dose is sufficient for matching ERT. **c,** Heatmap showing the proliferative cell dose (assuming that the dosed calles have 250 day half-life) against the GAA production rate of dosed cells, demonstrating that dose has a higher effect on therapeutic efficacy than GAA production gained from double correction. **d,** Heatmap showing that the GAA production against the half-life of the dosed cells (assuming that 250 million cells were dosed), indicating that the half-life of the dosed cells has a higher effect on therapeutic efficacy than the GAA production gained from double correction.

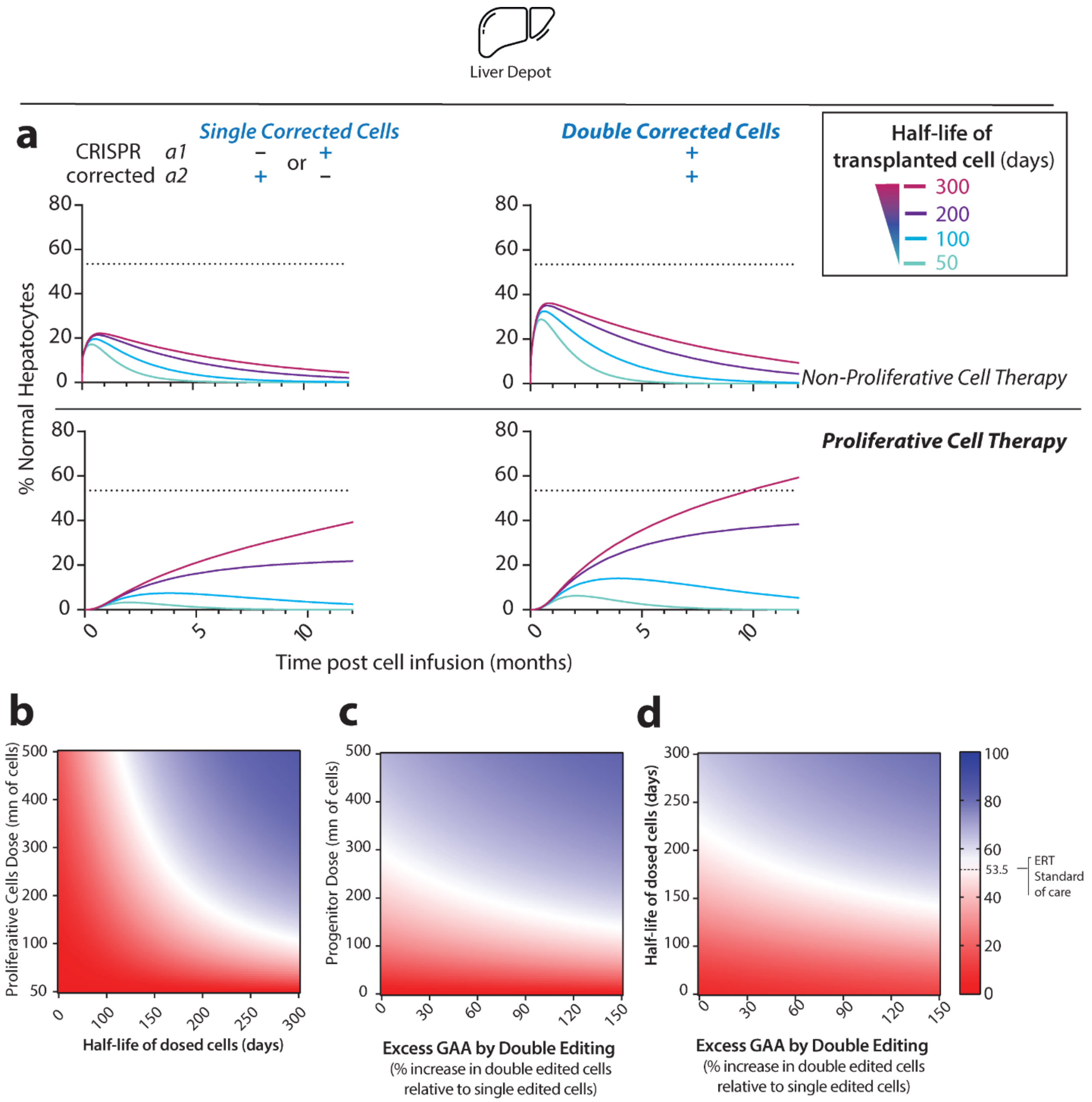

Supplementary Figure 15 | Cell therapy for Pompe disease requires persistent proliferative cells a, Plot showing the degree of phenotypically normal hepatocytes when either 10 billion non-proliferative single or double corrected cells are dosed or 100 million proliferative single or double corrected cells are dosed, , the double corrected cells whose GAA production is 150% excess relative to single corrected cells, for 4 different half-lives of dosed cells. For long lasting correction, proliferative progenitor cells need to have a half-life exceeding 100 days. **b,** Heatmap showing the proliferative cell dose (assuming double corrected cells with 75% excess GAA production relative to single corrected cells) against the half-life of dosed cells. If the dosed cells have a half-life similar to endogenous hepatocytes, a 75 million cell dose is sufficient for matching ERT. **c,** Heatmap showing the proliferative cell dose (assuming that the dosed calles have 250 day half-life) against the GAA production rate of dosed cells, demonstrating that dose has a higher effect on therapeutic efficacy than GAA production gained from double correction. **d,** Heatmap showing that the GAA production against the half-life of the dosed cells (assuming that 250 million cells were dosed), indicating that the half-life of the dosed cells has a higher effect on therapeutic efficacy than the GAA production gained from double correction.

Supplementary Notes

Comparison of gene correction therapy to existing therapies in development and standard of care

ERT is currently the only approved clinical treatment for Pompe disease[1,2]. However, patients require high levels of enzyme injected biweekly, rendering the treatment expensive and inconvenient[3]. ERT may also be less effective in a subset of patients that are cross-reactive immunologic material (CRIM) negative[4]. Therefore as an alternative, gene augmentation therapy by viral overexpression has long been assumed to be the most practical approach to genome surgery to address autosomal recessive disorders, especially compound heterozygous cases. Gene therapies have primarily made use of integrated viral cassettes including transgenes to express a normal GAA coding sequence from exogenous promoters. Viral gene therapy particles are injected either directly into muscle or administered systemically and transported to the liver. Infected cells can generate phenotype rescue (10-fold reduction in glycogen content), but fail to correct many non-transduced cells. These therapies also require high viral loads (>10^{10} viral genomes/kg)[5–8]. Silencing of the viral transgene, immune response to the viral vector, and insertional oncogenesis are outstanding concerns with these viral gene therapy approaches. Anti-sense oligonucleotides can also be introduced to correct splicing in diseased patients that possess mutations at splicing sites, but would only be beneficial to a subset of potential patients[9]. Finally, autologous cell therapy has been proposed using cells engineered to constitutively overexpress GAA[10]. None of these approaches retain endogenous regulation and levels of GAA.

Gene editing of primary fibroblasts from Pompe diseased patients

Patient-derived, primary fibroblasts with different monoallelic mutations in *GAA* were edited using S1mplexes to demonstrate editing and correction for multiple mutations. For each line, a mutation specific guide and a correction ssODN with codon wobble was designed and generated. Correction and editing efficiencies, as well as editor precision, are shown in **Fig. 5g,h**.

Isolation of gene-corrected iPSCs

We designed sgRNAs that were specific to the mutant allele by only evaluating those that contained the mutant site within the seed region of the sgRNA[18]. We exploited a RNA aptamer based approach developed in our lab, the S1mplex[19], that was previously demonstrated to promote precise correction of alleles at many loci, including the *GAA* locus. After designing S1mplexes to correct each of these mutated alleles (**Supplementary Tables 1-2**), we delivered the S1mplexes to the iPSCs.

As identifying double-corrected cells can be challenging, we complexed the specific sgRNA and ssODN pairs together and included a fluorophore as a marker for the presence of S1mplexes within the nucleus. Using a high-content analysis platform developed in our lab, ArrayEdit[11], we were able to enrich for properly edited iPSCs. On day one post plating we measured the presence of S1mplex within the nucleus as well as identified μFeatures that contained only one cell to ensure clonal populations. On days two through six we measured the number of cells to obtain a growth rate via day over day change. Finally, on day 7 we measured the number of cells as well as stained cells with LysoSensor to identify populations that may have been edited using phenotypic recovery as a marker. We also mock transfected WA09 and Pompe iPSCs and plated them on ArrayEdit and subjected them to the same high content analysis as a control. After 7 days we plotted each individual μFeature by its LysoSensor intensity and growth rate and color coded each feature by the presence of S1mplexes on day 1, (**Supplementary Figure 1c and 1d**). We observed a large population of clones that grew slower than the slowest mock transfected Pompe colonies suggesting that these populations maybe undergoing editing events. By comparing LysoSensor intensity we also observed that many of the μFeatures within the wells had higher, and therefore more acidic organelles than mock transfected Pompe iPSCs. Remarkably, many of the μFeatures had similar intensities to control WA09 hPSC colonies. By combining these data with the presence of S1mplexes we were able to select colonies that were potentially preferably edited at either loci individually or both simultaneously. Using this knowledge, we selected colonies of interest for expansion and analysis.

Following expansion of selected clones, we analyzed each one at both loci for the correction of mutations. We also designed the introduction of a PAM codon wobble to ensure that supplied donor DNA was the source for repair. When we looked at colonies that only had the presence of one S1mplex on day 1 we obtained clones that were edited at the specified allele. Interestingly, we did not isolate any clones that had indel mutations caused by NHEJ. Further, colonies that were positive for one S1mplex were not observed to be edited at the other locus. We next analyzed clones positive for both S1mplexes and managed to obtain a clone that was edited at both alleles and also contained the PAM wobble. There was also one colony that contained one PAM mutation while the other allele was repaired but did not introduce the PAM wobble. Importantly, across all screened clones we did not obtain any that contained indel products. We then selected one clone from each population (edited at either allele individually, or both) to assay for phenotypic recovery.

Edited mRNA transcript analysis

To analyze expression from the edited alleles, we performed deep sequencing of mRNA from the edited iPSC lines. In the unedited line we saw that at both loci, there was approximately a 1:1 balance in expression from the diseased and healthy alleles (**Supplementary Figure 6b-c**). This was predicted, as mRNA from both mutant alleles are expected to be expressed and similarly regulated. We then looked at the single corrected lines and observed that the corrected locus was expressed at a higher level (~5-fold increase) than the corresponding unedited locus (**Supplementary Figure 6b-c**). This finding was supported by conducting deep sequencing at both edited loci, as sequence data can be coupled to form a single mRNA transcript. Finally, when we analyzed the double corrected cells, we observed data similar to that of the unedited line in that both alleles are similarly expressed (**Supplementary Figure 6b-c**).

Glycogen clearance with edited cells

After confirming the molecular biology signature of the corrected lines, we explored the ability of the edited cells to clear glycogen from the lysosome, signifying a return to a normal phenotype. First, we obtained protein lysates from all iPSC lines and incubated them with 4-methylumbelliferyl-β-galactopyranoside (4-MUG). Active GAA protein cleaves this substrate and releases a fluorophore that can be detected with a standard plate reader. Consistent with previous results, we observed that the unedited cells had minimal GAA activity. Interestingly, when activity of the single and double corrected lines was measured, all lines had approximately the same cleavage ability. This is consistent with the lack of symptom presentation in Pompe disease carriers. As an internal assay control, we also measured neutral GAA activity and saw no difference between any of the lines, including the unedited cells (**Supplementary Figure 7b,c and Supplementary Figure 8c,e**). More importantly, we also tested the GAA activity of spent media after 24 hours of culture (**Supplementary Figure 8e**).

In silico **model development**

The *in silico* model was constructed by considering four categories of mechanisms in a variety of cells and tissues:

1. Progenitor growth and differentiation
2. GAA absorption and production to generate transient enzymatically cross-corrected phenotypes
3. Genome editing – precise and imprecise editing at either or both alleles
4. Enzymatic cross-correction of distal tissues from depot – cardiac muscle and skeletal muscle

As described below, simplified differential equation models for each of these four mechanisms were assembled to generate the overarching model for Pompe disease.

Mechanism 1: Progenitor Growth and Differentiation

Infantile liver growth was modeled to show the propagation of early gene editing over time. Since the infant liver grows through the proliferation and differentiation of a non-abundant progenitor, editing the

progenitor has profound effects on the phenotype of the mature cells after a certain period of growth. This model assumes that mature hepatocytes in the uninjured liver are unlikely to be proliferative[12], and that there exists a hepatocyte progenitor, which divides and gives rise to non-proliferative mature hepatocyte[12]. These progenitors are shown to occupy approximately 1% of the liver [12].

Liver growth is significant in the first year of life, the 50th percentile weight of a newborn liver is 140 g, and at 1 year, its weight is 400 g[13]. The number of hepatocytes in an adult has been estimated to be 2.4×10^{11} [14], and the corresponding mass of the liver is about 1561 g. Therefore, the number of hepatocytes in the newborn liver is estimated to be 6.14×10^{10}.

The following equations were used to describe the growth and maturation of hepatocyte progenitor cells in the uninjured liver. Hepatocyte progenitor cells indicated by *P*, and mature non-proliferative hepatocytes indicated by *M*. The ratio of mature:progenitor hepatocytes is represented as χ: 1. The rate of proliferation of progenitor *P* is indicated by λ and the rate of differentiation of progenitor *P* is indicated by γ.

$$P \rightarrow M \tag{1}$$

$$P \rightarrow P + P \tag{2}$$

$$M = \chi P \tag{3}$$

$$\frac{dP}{dt} = \lambda P - \gamma P \tag{4}$$

$$\frac{dM}{dt} = \gamma P \tag{5}$$

The initial and boundary conditions utilized to solve these equations were $P = 6.08\times10^8$, $M = 6.08\times10^{10}$ at time t = 0, and $P = 1.75\times10^9$, $M = 1.73\times10^{11}$ at time t = 365 days. It is also assumed that the progenitor:mature cell ratio remains 1:100 through this period of growth. Solving equation (3) – (5) for the parameters analytically, λ and γ were found to be 0.2902 d^{-1} and 0.2876 d^{-1} respectively.

Mechanism 2: Estimation of GAA Absorption and Production by Healthy Cells

GAA produced by striated muscle tissue and the liver is secreted extracellularly and can be detected in serum. Therefore, to model GAA absorption and production, it is necessary to estimate both the quantity of GAA present in healthy infants and approximate excess secreted GAA. Production of glucose within the body happens through glycogenolysis and gluconeogenesis[15]. Tracking glucose production within the body from stored glycogen stores (glycogenesis) can be utilized to estimate the amount of GAA in the body.

The rate of glycogenolysis in neonates is 0.4 – 0.9 mg kg^{-1} min^{-1}. Therefore we used the lower estimate of 0.4 mg kg^{-1} min^{-1}, or 1.708×10^{3} μmol h^{-1} L^{-1} to calculate the quantity of GAA required to correct an unedited cell. The calculation assumes that the activity is measured as a rate of glucose production, in a newborn weighing 3.5 kg, and a blood volume of 273 mL[16]. This was used along with the correlation between GAA activity and GAA protein mass[17], to estimate that the total mass of GAA protein within the body to 11.99 mg, or 1.12×10^{6} molecules of GAA per WT healthy cell (containing two correct alleles). Therefore, 1.12×10^{6} molecules of GAA are required to correct one unedited cell. Using the difference between the minimum and maximum glycogenolysis rate and the half-life of cellular GAA to be 7 days[18], the excess GAA production rate (beyond the required amount for cellular function) was estimated to be 9.702×10^{5} molecules $cell^{-1}$ d^{-1} for a cell with two correct alleles.

Cross-correction of cells via external GAA and cross-corrected cells reverting were modeled using three equations, the first representing the cross-correction of *P* to *PCC* which is enzymatically catalyzed by GAA, the second representing the decay of cellular GAA, represented as the conversion of *PCC* to *P*, and the third, representing the decay of GAA.

$$P \rightarrow PCC; GAA \qquad (6)$$

$$PCC \rightarrow P \qquad (7)$$

$$GAA \rightarrow \emptyset \quad (8)$$

$$\frac{dP}{dt} = -(k_{CC})(GAA)P + (k_d)PCC \quad (9)$$

$$\frac{dPCC}{dt} = (k_{CC})(GAA)P - (k_d)PCC \quad (10)$$

$$\frac{dGAA}{dt} = -k_{GAA}GAA \quad (11)$$

$$\emptyset \rightarrow GAA; P_{Correct} \quad (12)$$

$$\frac{dGAA}{dt} = -k_{SecGAA}P_{Correct} \quad (13)$$

Using a pseudo steady-state assumption and assuming 8,594×10^{16} molecules are dosed (enough GAA for full correction of all hepatocytes), along with assuming 50% correction in 0.5 days, the value of k_{CC} was evaluated to be 1.613×10^{-17} GAA^{-1} d^{-1}. Using the half-lives stated above k_d was estimated to be 0.0990 d^{-1}, and k_{GAA} was estimated to be 4.158 d^{-1}. Finally, using the GAA production rate calculated above, k_{SecGAA} was set to 9.702 ×10^5 molecules $cell^{-1}$ d^{-1} for a cell with two correct alleles and 4.851 ×10^5 molecules $cell^{-1}$ d^{-1} for a cell with a single corrected allele.

Mechanism 3: Gene Editing – Monoallelic Gene Editing

In order to model gene editing at a single allele, two models were combined, a central dogma model and a gene editing model. The first was a model of the central dogma[19], which was utilized to generate the effective RNP dose from plasmid, mRNA or AAV dosage. This model was combined with the model for liver progenitor growth and differentiation (Equations (1) – (5), adapted for mice, so number of hepatocytes 9.3771×10^7, λ was 0.5346 day^{-1}, γ was 0.5293 day^{-1}), described above. To make the model specific to $FAH^{-/-}$ mice, cell death was modeled such that removal of NTBC caused a 20% loss of hepatocytes within 30 days[20], k_{death} was 0.0074 day^{-1}, and T_{NTBC} was 1 when NTBC was removed. This was combined with the model described below for gene editing and modified for different delivery methods.

$$P \rightarrow null \tag{14}$$

$$M \rightarrow null \tag{15}$$

$$\frac{dP}{dt} = -k_{death} \times T_{NTBC} \times P \tag{16}$$

$$\frac{dM}{dt} = -k_{death} \times T_{NTBC} \times M \tag{17}$$

The following equations were used to model the editing of progenitor *P* at allele 1 using *S1*, a genome editor targeting allele 1 to form precisely edited progenitor *P1* and imprecisely edited progenitor *P1′*. The rate of precise editing is $k1$ and of imprecise editing is $k1'$. The rate of genome editor decay is k_{sd}.

$$P + S1 \rightarrow P1 + P1' \tag{18}$$

$$S1 \rightarrow \emptyset \tag{19}$$

$$\frac{dP}{dt} = -(k1 + k1')(S1)P \tag{20}$$

$$\frac{dP1}{dt} = (k1)(S1)P \tag{21}$$

$$\frac{dP'}{dt} = (k1')(S1)P \tag{22}$$

$$\frac{dS1}{dt} = -k_{sd}S1 \tag{23}$$

The combined model was then modified for the following delivery methods.

For plasmid delivery of *Spy*Cas9[20], the initial DNA dose used was 3.411×10^{12} molecules corresponding to 30 μg DNA injection of a 8000 bp plasmid. At times greater than 3 days, T_{NTBC} was made 1. The precise to imprecise ratio used was 0.3396 based on editing data provided in the study. The editing outcomes were assayed on day 33 post treatment in this study.

For plasmid delivery of RA6.3 base editor[21], the initial DNA dose used was 6.54×10^{12} molecules corresponding to 60 µg DNA injection of a 8400 bp plasmid. At times greater than 6 days, T_{NTBC} was made 1. The precise to imprecise ratio used was 0.9 based on editing data provided in the study. The editing outcomes were assayed on day 33 post treatment in this study.

For mRNA delivery of *Spy*Cas9 and AAV delivery of the HDR template[22], it was assumed that the HDR template is in stoichiometric excess to the *Spy*Cas9, and therefore, HDR availability was not independently modeled. The mRNA dose was used was 1.65×10^{13} molecules corresponding to 40 µg (about 2 mg/kg of a 10 week mouse with a weight of 20g) of a 4500 bp mRNA. The data provided in the paper indicated a modified RNA degradation rate of 2.49 day^{-1}. At times greater than 7 days, T_{NTBC} was made 1 to compare to other studies. The precise to imprecise ratio used was 0.0320 based on editing data provided in the study. The editing outcomes were assayed on day 7 post treatment in this study.

For AAV delivery of *Sau*Cas9 to correct gene *Otc*[23], the AAV was modeled as an effective DNA dose of 50 copies per cell, based on data provided in the study, or 2.843×10^{9} molecules. The DNA decay rate was modeled based on the decay in the number of copies of *Sau*Cas9/cell as reported, and was 0.0553 day^{-1}. This DNA decay rate is an order of magnitude lower than what was reported in the central dogma model. At times greater than 14 days, T_{NTBC} was made 1. The effective DNA dose is assumed to have taken place 1 week post virus introduction, based on data presented in the study. The precise to imprecise ratio used was 0.3226 based on editing data provided in the study. The editing outcomes were assayed on day 21 post treatment in this study, in our simulation, it is represented as day 14, as we assumed that the viral transfection and DNA expression took a week.

For the training and validation, bootstrapping was used to generate 10,000 replicates based on the published data for each of the gene correction publications described above[20–23] on the percentage of precise edits in

the liver. For each bootstrapped percentage edit, the corresponding total editing rate and corresponding precise ($k1$) and imprecise ($k1'$) editing rate constants were evaluated using model iteration.

For each set of editing rate constant, the rate of liver genome editing was evaluated by calculating the flux of the gene editing process [equations (21) and (22)] normalized against the total number of cells against the liver at each time point. This flux was integrated over the time period of the model to evaluate the time averaged rate of change of liver genome. From the 4×10^4 bootstrapped replicates, the mean, and mean ± standard deviation of the time averaged rate of change of liver genome was evaluated. These were divided by the RNP dose[24] to generate the second order editing rate constant ($k1'$).

For *Fah* substitution via microhomology mediated editing[25], NTBC withdrawal was programmed to be on day 7, and the simulation validation time point was day 30. Model precision was set to 30%, based on their reported flow data.

For *Hpd* editing via plasmid delivery of *Nme*Cas9[26], the DNA dose was recalculated using the size of the *Nme*Cas9 plasmid, as it was smaller (4377 kb) than pX330 used by both the plasmid *Spy*Cas9 and MMEJ studies. Simulation was run to 43 days to compare results. The imprecise editing rate constant was set to zero as all edits lead to cells that are metabolically reprogrammed and are immune to death due to NTBC withdrawal. An event was added at 21 days to simulate NTBC supplementation for 3 days as per the experimental protocol. The progenitor growth and differentiation rates were altered, as the mice in this study were between 15 – 20 weeks old, as opposed to 8 weeks old.

For *Icam-2* editing using mRNA lipid nanoparticles[27], the molar dose of *Spy*Cas9was calculated based on the 1:1 *Spy*Cas9 mRNA:sgRNA dose, and it was assumed that the *Spy*Cas9 is the limiting reagent. There

was no selection event, as this model does not select for edited phenotypes, and the precise editing rate constant was set to zero, as this study only looks at indel generation and not precise gene correction.

For *Pah* targeting using an intein split base editor delivered using two AAVs[28], the AAV dose was scaled using data from the *Otc* editing story[23]. Precision was set to 64.52% based on the published number of alleles with the desired base substitution (precisely edited) compared to the number of residues proximal to the cut site with undesired base substitution (imprecisely edited).

From the 4 x 10^4 bootstrapped replicates, the mean, and mean ± standard deviation of the time averaged rate of change of liver genome was evaluated. These were divided by the human RNP dose to generate the second order editing rate constant $(k1 + k1')$. The RNP per cell used in the *Pten* editing study was multiplied with an estimate of the total number of hepatocytes in the newborn human liver to calculate the human RNP dose. With this second order single editing rate constant calculation, the third order single double editing (biallelic editing) rate constant was calculated. The second order single editing rate constant range was evaluated to be 4.23×10^{-21} to 3.01×10^{-20} $s1mplex^{-1}$ day^{-1}.

Mechanism 3: Gene Editing – Biallelic Gene Editing

Biallelic gene editing was hypothesized to occur in one of two ways – sequentially (without co-CRISPR-cooperation[29]) and simultaneously with both S1mplexes cooperating to edit at both alleles at the same time (with co-CRISPR-cooperation). The co-CRISPR-cooperation is represented by a coefficient, the co-CRISPR-coefficient k_{CCC}. When $k_{CCC} = 1$, the rate of sequential biallelic editing should be approximately equal to the rate of simultaneous biallelic editing. If $k_{CCC} > 1$, editing on one allele should make editing on the other allele easier (higher rate); and, if $k_{CCC} < 1$, editing on one allele should make editing on the other allele more difficult (slower).

In order to develop the mathematical expressions that satisfy the above assumptions, two mathematical models were constructed in MATLAB Simbiology (R2020a). The first model constructed which describes sequential editing of progenitor *P* at allele 1 by S1mplex *S1* forming *P1* followed by editing of *P1* at allele 2 by S1mplex *S2* forming *P12*.

$$P + S1 \rightarrow P1 \tag{20}$$

$$P1 + S2 \rightarrow P12 \tag{21}$$

$$S1 \rightarrow \emptyset \tag{22}$$

$$S2 \rightarrow \emptyset \tag{23}$$

$$\frac{dP}{dt} = -(k1)(S1)P \tag{24}$$

$$\frac{dP1}{dt} = (k1)(S1)P - (k2)(S2)P1 \tag{25}$$

$$\frac{dP12}{dt} = (k2)(S2)P1 \tag{26}$$

$$\frac{dS1}{dt} = -k_{sd1}S1 \tag{27}$$

$$\frac{dS2}{dt} = -k_{sd2}S2 \tag{28}$$

The second model constructed describes simultaneous biallelic editing of progenitor *P* at both alleles to form *P12* in a single step.

$$P + S1 + S2 \rightarrow P12 \tag{29}$$

$$P1 + S2 \rightarrow P12 \tag{30}$$

$$S1 \rightarrow \emptyset \tag{31}$$

$$S2 \rightarrow \emptyset \tag{32}$$

$$\frac{dP}{dt} = -(k12)(S1)(S2)P \tag{33}$$

$$\frac{dP12}{dt} = (k12)(S1)(S2)P \tag{34}$$

$$\frac{dS1}{dt} = -k_{sd1}S1 \tag{35}$$

$$\frac{dS2}{dt} = -k_{sd2}S2 \tag{36}$$

These first approximation is based on the harmonic mean of $k1$ and $k2$. The harmonic mean is utilized typically when rates of processes need to be averaged. The units of $k1$ and $k2$ are S1mplex^{-1}d^{-1}, and the units of $k12$ are S1mplex^{-2}d^{-1}. All S1mplex concentrations used in the evaluation of these rate constants are based on the initial S1mplex concentrations dosed.

$$k12 = k_{CCC}\frac{(k1)(k2)}{(k1)+(k2)} \cdot \frac{1}{(S1)+(S2)} \tag{37}$$

The double editing rate constant range was evaluated to be 2.10×10^{-40} to 1.06×10^{-38} s1mplex^{-2} day^{-1}.

Mechanism 4: Enzymatic cross-correction of distal tissues from liver depot – cardiac muscle

Modeling the GAA consumption of the heart was performed by first estimating the GAA activity in the healthy newborn heart, and then scaling the GAA requirement for cross-correction by the growth rate of the heart. We assume that the GAA activity of the heart and correspondingly, the GAA requirement for enzymatic cross-correction scales with heart tissue size, scales with both muscle enlargement and division [30]. The number of cardiomyocytes in the left ventricle increase from 1 – 2 billion in 5 years, while left ventricle volume increases from $2.5 – 15 \times 10^3$ μm^3 in 5 years [30]. Since, the percentage change in number of cells in the first year is 20%, and in cardiac volume is 400%, we consider changes in cardiac glycogen content based solely on cardiac muscle volume or mass. The heart weight of a newborn infant is approximately 25 g [31]. The GAA activity of the murine heart muscle has been reported to be 21.4 nmol/h/mg protein in healthy mice [32], and muscle tissue is approximately 15% protein by weight [33]. Finally, since mouse muscle cells have comparatively only 10% of the glycogen content as human muscle cells [34], we estimate that the GAA activity of human cardiac muscle to be approximately 10% of murine GAA activity,

2.14 nmol/h/mg. Using the correlation between GAA activity and weight [17], the GAA content of the newborn heart is 55 ng/mg protein. The initial volume of the heart is 25 g, and it grows at 400% linearly per year to 125 g in the first year.

Using a pseudo steady-state assumption and assuming 1.831×10^{15} molecules are dosed (enough GAA for full correction of all cardiac muscle cells), along with assuming 50% correction in 0.5 days, the value of k_{CC} was evaluated to be 7.571×10^{-16} GAA^{-1} d^{-1}. Using the half-lives stated above k_d was estimated to be 0.0990 d^{-1}. Given that ERT at 20 mg/kg raises murine GAA levels to 2000% of normal, but cardiac GAA levels to only 32% of normal [2], we model an additional tissue dosing factor for cardiac GAA (termed as a "loss factor" in the model code). Through preliminary simulations of rhGAA ERT in the model, the cardiac GAA dose factor was estimated to be 0.005, implying that only 0.5% of the rhGAA dosed in ERT reaches the heart at therapeutically beneficial GAA. For gene therapy this loss factor is estimated to be 400% higher as the immune response (anti-GAA antibody levels) to rhGAA produced through gene augmentation therapy is 80% lower than the immune response to rhGAA ERT [35].

Mechanism 4: Enzymatic cross-correction of distal tissues from liver depot – skeletal muscle

Modeling the GAA consumption of skeletal muscle tissue was performed by first estimating the GAA activity in healthy newborn skeletal tissue, and then scaling the GAA requirement for cross-correction by the growth rate of skeletal tissue. We assume that the GAA activity of skeletal muscle and correspondingly, the GAA requirement for enzymatic cross-correction scales with skeletal tissue size (i.e., scales with muscle enlargement rather than cell division as muscle cells are generally considered to be post-mitotic[36]). The GAA activity of the murine skeletal muscle has been reported to be 13.5 nmol/h/mg protein in healthy mice [32], and muscle tissue is approximately 15% protein by weight [33]. Finally, since mouse muscle cells have comparatively only 10% of the glycogen content as human muscle cells [34], we estimate the GAA activity of human striated muscle to be approximately 10% of murine GAA activity, 1.36 nmol/h/mg. Using the

correlation between GAA activity and weight [17], the GAA content of the newborn skeletal muscle is 35 ng/mg protein. Skeletal muscle mass represents about 25% of the muscle mass of an infant and is assumed to grow in proportion to the infant. Hence, the non-cardiac muscle mass at birth is assumed to be 850 g and at 1 year of age is assumed to be 2500 g.

Using a pseudo steady-state assumption and assuming 3.834×10^{15} molecules are dosed (enough GAA for full correction of all muscle tissue), along with assuming 50% correction in 0.5 days, the value of k_{CC} was evaluated to be 3.611×10^{-16} GAA^{-1} d^{-1}. Using the half-lives stated above k_d was estimated to be 0.0990 d^{-1}. Given that ERT at 100 mg/kg raises murine GAA levels to 2000% of normal, but skeletal muscle GAA levels to only 32% of normal [2], we model an additional tissue dosing factor for skeletal GAA (termed as a loss factor in the model code). Through preliminary simulations of rhGAA ERT in the model, the skeletal GAA dose factor was estimated to be 0.002, implying that only 0.2% of the rhGAA dosed in ERT reaches the skeletal muscle at therapeutically beneficial GAA. For gene therapy this loss factor is estimated to be 400% higher as the immune response (anti-GAA antibody levels) to rhGAA produced through gene augmentation therapy is 80% lower than the immune response to rhGAA ERT [35].

Assembly of the full in silico model

The model was generated using MATLAB scripts for generating the species and the reactions. The model was then edited in the SimBiology interface to add the doses of genome editor and rhGAA, along with rules for calculation of several rate equations. MATLAB R2020a was utilized for simulating the model using the ode15s solver. For the generation of the heatmaps sbioaccelerate was utilized to speed up execution of the model.

Global sensitivity analysis of the in silico model

Efficacy is most sensitive to tissue morphogenesis parameters (**Fig. 6e**) due to the nature of hepatocyte growth, where in a rare (1% abundance) progenitor gives rise to all new cells contributing to organ growth[12]. For parameters related to the design of genome editors, the efficiency and genome editor stability, followed by precision and progenitor affinity are cooperative (**Fig. 6f, 6g and 6h**), and engineering both genome editor performance and improving genome editor stability can both assist in greater phenotypic correction. The coCRISPR cooperativity sensitivity was 2 orders of magnitude lower than the other sensitivities, indicating that outcomes were relatively insensitive to the cooperativity between genome editors targeting multiple alleles within the same cell, the so called coCRISPR strategy. Double edited cells are orders of magnitude lower than single edited cells in the final distribution of genotypes (**Fig. 6d**), so high cooperativity ends up having a lower effect on rescue than the other efficiency, dosing and delivery mechanisms described in the main text. This result indicates that administration of genome editors attempting to correct different alleles need not necessarily be administered at the same time, and that alternating, sequential or staggered dosing would likely have similar efficacy, assuming that delivery, immune response or other factors are largely similar for each dose. There could be safety benefits to such approaches to reduce the likelihood of genomic deletions, translocations or inversions from multiple double strand breaks within the same cell.

Discussion on GETEM Model Assumptions

The model of for ERT uses clinical results from glycogenolysis studies to generate a per cell requirement for GAA in the human body, the dosing regimen for ERT is from a different unconnected clinical study. This glycogenolysis study compared two methods for measuring glycogenolysis and gluconeogenesis in premature infants[15]. We then assume that the per-cell requirement for GAA in these infants is similar to that of normal neonates and infants. Similar studies have not been conducted for older healthier infants and children. Therefore, we also investigated the sensitivity of liver GAA requirement (**Fig. 6e**) and conclude

that even if the per-cell GAA requirement and production estimates are inaccurate, tissue morphogenesis would still play the major role in genome editing therapy.

GETEM predicts a high amount of GAA being produced in the liver, but only a small proportion of this GAA being therapeutically available to striated cardiac and skeletal tissue (2% and 0.8% respectively for gene corrected GAA). These numbers are based on pre-clinical studies of lysosomal storage disorders[32]. While ongoing literature on GAA gene augmentation therapy acknowledges that despite extremely high levels of exogenous GAA produced in the liver (2030% normal), the GAA concentrations in heart (205% normal) and skeletal muscle (51.2% - 100% normal, depending on the muscle) are significantly lower. Recent studies have acknowledged some methods to address this issue[37], including treatment with long acting β2-agonist clenbutrol to increase cellular uptake of GAA via the cation-independent Mannose Phosphate Receptor (CI-MPR), along with gene therapy. The methods used for increasing gene augmentation therapy efficacy can be successfully applied to the described genome editing therapy model to increase its efficacy, as we demonstrate by simulating a GAA stabilizing adjuvant dosed in conjunction with genome editing therapy (**Fig. 6i**). Understanding the reasons for GAA loss between the liver and distal organs will aid the development of more effective therapies; mathematically accounting for this loss opens underexplored avenues for improving gene therapy outcomes.

The dosing in the mathematical model is based on the amount of RNP directly reaching the liver. These estimates were derived from data based on RNP dosing in mouse livers[24]. However, a significant portion is likely to reach the liver, as nanoparticle pharmacokinetics studies have indicated that the liver is a destination for sequestration of nanoparticles[38], thus with some scaling our model findings stand. Our modeling of multiple doses compared to a single large dose (**Supplementary Figure 13**) appears to be counterintuitive, as the large dose appears to provide equivalent effect regardless of the age of administration. This is likely due to high sensitivity of the tissue morphogenesis factors, which mask the

effect of increased therapeutic dose on the percentage normal cardiac tissue. For cell therapy, the cell dose is less sensitive than the persistence of the cells dosed (**Fig. 7e**) and tissue morphogenesis factors.

Finally, with regards to cell therapy, we have demonstrated quantitatively the importance of persistence and proliferative capacity of the cells dosed. This model assumes the number of cells that successfully functionally engraft, which is likely to be lower than the total number of cells dosed. Additionally, the model assumes that the engrafted cells behave similar to native progenitor and mature liver cells with respect to growth and differentiation characteristics. Studies that have looked at xenogenic progenitor cell dosing (human $PDX1^+$ pancreatic progenitors in mice) have shown that once properly purified, the progenitor cells grow at rates similar to endogenous tissue and differentiate *in vivo* into terminal non-proliferative cells[39].

Similar implantation studies performed in the liver also show that human liver cell populations containing progenitors can repopulate mouse livers without tumor formation or fibrosis[40]. Therefore, the assumption that engrafted cells will behave similar to endogenous tissue is justified.

Death rates were not considered for the genome editing model, they were intrinsically included in the growth rate. For the implanted cells, the death rate was only implemented in the dosed progenitor population. Once these cells differentiated into cells resembling mature hepatocytes, they added to the mature hepatocyte population in the model. These edited mature hepatocytes are assumed to continue to remain in the liver during the duration of therapy modeled (12 months). We assume that the persistence phenotype modeled applied only to dosed progenitors and their undifferentiated progeny, their differentiated progeny was assumed not to have this phenotype. While several assumptions were made to model ERT, genome editing and cell therapy in GETEM, they are grounded in the current *in vivo* understanding of these phenomena.

Finally, all the modeling of growth of liver cells has been done assuming linear growth of a male infant at the 50th percentile during the first 15 months of age[41]. Our model would have to be modified beyond this time through the analytical solution of Equations 1 – 5, as the growth rates and characteristics change after the first year of growth. Due to this limitation, all results shown only consider growth until this timepoint. This limitation is clinically relevant because the median lifespan of patients diagnosed with infantile onset Pompe disease is 8.7 months without treatment[42]. Our model shows that genome editing and cell therapy can provide durable responses within this timeframe without continued dosing beyond this timeframe, as required for ERT.

Supplementary Video Files

Pompe_backupgif.gif : Graphics Interchange Format video demonstrating the spontaneous beating of unedited Pompe disease iPSC derived cardiomyocytes.

746_backupgif.gif : Graphics Interchange Format video demonstrating the spontaneous beating of 746 Correct iPSC derived cardiomyocytes.

dTgif.gif : Graphics Interchange Format video demonstrating the spontaneous beating of dT Correct iPSC derived cardiomyocytes.

Doublegif.gif : Graphics Interchange Format video demonstrating the spontaneous beating of DoubleCorrect.c1 iPSC derived cardiomyocytes.

Supplementary Tables

Supplementary Table 1: Protospacer and respective PAMs used for genomic targeting.

Name	Protospacer	PAM
1441delT sgRNA	CTCGTTGTCCAGGTAGGCCC	GGG
2237G>A sgRNA	TGGACCACCAGCTCCTGTAG	GGG
W746X sgRNA	GGACCACCAGCTCCTGTAGG	GGG
D645N sgRNA	GCCCAGGAAGCCGCAGACGT	TGG
R660H sgRNA	CAGAGGAGCTGTGTGTGCAC	TGG

Supplementary Table 2: ssODNs used to direct gene correction after double strand break formation.

**Name**	**Sequence (5`-3`)**
1441insT ssODN	CTTCCATGCAGGCCCTGGGTGGGGCCGGGTCTCCCCACTGCAGC<br>CTCTCGTTGTCCAGGTATGGCCCGGATCCACTGCCTTCCCCGACTTCACCAACCCC
2237A>G ssODN	TGCCCATCCCCCTTGCAGGTTCCCCAAGGACTCTAGCACCTGGA<br>CTGTGGACCACCAGCTCCTGTGGGGAGAGGCCCTGCTCATCACCCCAGTGCTCCAG
W746X ssODN	TGCCCATCCCCCTTGCAGGTTCCCCAAGGACTCTAGCACCTGGACTGTG<br>GACCACCAGCTCCTGTGGGGAGAGGCCCTGCTCATCACCCCAGTGCTCCAG
D645N ssODN	AGAAATCCTGCAGTTTAACCTGCTGGGGGTGCCTCTGGTCGGGGCAGACGTCT<br>GTGGCTTCCTGGGCAACACCTCAGAGGAGCTGTGTGTGCACTGGACC
R660H ssODN	GGGCCAACGTCTGCGGCTTCCTGGGCAACACCTCAGAGGAGCTATGTGTGCGCTG<br>GACCCAGCTGGGGGCCTTCTACCCCTTCATGCGGAACCACAACAG

Supplementary Table 3: Forward and reverse primers for genomic loci.

Name	Primer F (5`-3`)	Primer R (5`-3`)
1441delT genomic	AGCTGCTCATTGACCTCCAG	CAATCCACATGCCGTCGAAG
2237G>A genomic	AATTCAGCCTCTTCCTGTGC	CATACGTTCCTCTTTCCGCC
Full length genomic	TGACAGGTTTCCCTCTTCCCAG	TTGATAACCTACACTGCGGGGG
1441delT qPCR/NGS	AGTGGGGCTTCCATGCAG	GGTTGGTGAAGTCGGGGAAG
2237G>A qPCR/NGS	CCAAGGACTCTAGCACCTGGAC	GGGAAGTAGCCAGTCACTTCGG
W746X NGS	TCCCATTCATCACCCGTATGC	AGGTCGTACCATGTGCCCAA
D645N R660H NGS	CTGAGGACCAGCCTGACTCT	CCACCCTACCAGACTGAGCA

Supplementary Table 4: Off-target sequences and corresponding genomic locus for each sgRNA used. Mismatches from protospacer are labelled in red.

sgRNA Target Sequence	Off-target sequence		Chromosome location
2237G>A<br>TGGACCACCAGCTCCTGTAG	OT1	TAGACCACCAGCTCCTGCAG	chr8:-42696136
	OT2	CTCACCACCTGCTCCTGTAG	chr9:-123379574
	OT3	TTGACCAGCAGCTCCTGTCG	chr15:-77699091
	OT4	TGGCACACCAGCTCCTGTTG	chr14:-58627302
	OT5	GTGACCAAGAGCTCCTGTAG	chr17:-8010424
	OT6	AGGGTCACCACCTCCTGTAG	chr10:-71293697
	OT7	TCTACAAGCAGCTCCTGTAG	chr4:+19857056
	OT8	TGGTGCACAGGCTCCTGTAG	chr18:-10682896
	OT9	AGCACCACCAGCTCCTGCAG	chr14:-73799987
	OT10	TGGGCCATCTTCTCCTGTAG	chrX:+64921874
1441delT<br>TGGACCACCAGCTCCTGTAG	OT1	CTGGTTGTCCAGGTGGGCCC	chr19:+9976610
	OT2	CTCGATGGCCAGGTAGGCCT	chr9:+113788274
	OT3	ATTATTGACCAGGTAGGCCC	chr20:-42195228
	OT4	CTACCTGCCCAGGTAGGCCC	chr12:-120926049
	OT5	CTCCTTGCCCAGGTAGCCCC	chr3:+13655121
	OT6	CTTCTTGTCCAGGTAGGCAC	chr22:-21693839
	OT7	CACGTGGTCCAGGTAGGCCT	chr14:-104755666
	OT8	CTGGCTGACCAGGTAGGCCT	chr11:-110492658
	OT9	CGCATTCTCCAGGTAGGCCA	chrX:+71130220
	OT10	CTCCACCTCCAGGTAGGCCC	chr18:-15005864

Supplementary Table 5: Forward and reverse primers used to amplify off-target genomic loci.

Off-Target Primer	Primer F (5`-3`)	Primer R (5`-3`)
2237-OT1	CCCTCCTCTGTGTGCCATTA	GTGCCATATTTTGGGGACCAC
2237-OT2	GGGGCATGGTCAGATGATGG	CACAGAAATTCCTGAGGCCAAC
2237-OT3	GGAGAGGCTGACCTTCATGG	TCGTGCTTTCCTGACCATCG
2237-OT4	CCTGGGGGAAAGGTAAAAGA	GCTGGAATGGTCTCGATCTC
2237-OT5	CACCCTGGAGTAGGCTTTCA	AGGACAGTGCCCTCACAATC
2237-OT6	ATGACCTCTGGGAATGCAGA	TCTGATGCTCCCTCAGTCCT
2237-OT7	CCCATTGCAACTGTGAACAA	TCTCCAACCATCAAGGAACA
2237-OT8	TCGCTCTGAAATGGGGATAG	CATATTTCCGGCACCATCTT
2237-OT9	CGTGTCTAGCCCCATCTCTT	CCTCCTCCCTGGTCCTAAAC
2237-OT10	GGGGAGCTTACCACCTTAGC	GCCTCTGTCTTCCAAATTGC
1441-OT1	AGTGTGCTTCCACTGTCGTT	GTGCGGGTAACCTTCTCCAT
1441-OT2	TTCCTCTGCTGCTGAGTTGG	GCCGATTAAAAGGCTGTCGC
1441-OT3	AGAGCCCTGGAGGTCATTGT	CTGTCTGGCCTCTGAATCGG
1441-OT4	ATTAGCCGGTGCCATGATAA	CGAGGAGCAACAGGGTAGAG
1441-OT5	GGGCAGTGTACCAGGTTAGG	GGTCCCCTAGGGTTAGCTTG
1441-OT6	CTGGACGACCTGAGCATTTT	AGAGTTCCACTTGGCCAGAA
1441-OT7	CATTCCACAGAGCCTGGTTT	ACTGCATAGGGATGGGACTG
1441-OT8	CACTTTCCCCAGCTCTTCAG	ATGGCACACCTGGTCCTAAC
1441-OT9	TCTCCACACGTGTTCCAATC	AAATATACACGGCCCACACC
1441-OT10	CTGTCCTCAGCCAACAGTGA	GTGTTCACAGCCAACACAGG

Supplementary Table 6: Subset of mutations in *GAA* and accompanying allele-specific sgRNAs. Red denotes location of mutations relative to wildtype for allele-specific sgRNAs.

Mutation	sgRNA Sequence	Allele Frequency $(x10^{-5})$
c.118C>T	GAGGAGCCACTCAGCTCTCAGGG	0.86
c.258dupC	ATCGAAGCGGCTGTTGGGGGGGG	2.65
c.525delT	CTGGACGTGATGATGGAGAC-GAG	7.04
c.1822C>T	AGTGGCCGGCGTATCAGCCGTGG	2.76
c.1827delC	TGCTGGCCACGGCCGATA-GCCGG	3.75
c.1930_1936dupGCCGACG	AAGCCGCAGACGTCGGCCGTCGG	1.17
c.2242dupG	CACCAGCTCCTGTAGGGGGGAGG	1.66
c.2560C>T	ACCAAGGGTGGGGAGGCCTGAGG	21.5
c.2662G>T	TAACACGATCGTGAATTAGCTGG	1.65

Supplementary Table 7: Numbers of lysosomes analyzed for determination of enzymatic cross correction in **Fig 4**.

**Media Source**	**a1**	**a2**	**n**
Pompe iPSC Cardiomyocytes Conditioned Media	–	–	262
1441delT Correct Cardiomyocytes Conditioned Media	–	+	201
2237G>A Correct Cardiomyocytes Conditioned Media	+	–	261
Double Correct.c1 Cardiomyocytes Conditioned Media	+	+	249
Control hPSC line Cardiomyocytes Conditioned Media	+	+	135
Fresh media + 10 nM rhGAA	N/A	N/A	302

Supplementary Table 8: Published data for training and validating GETEM.

**Gene Therapy Modality Being Modeled**	**Number of Mice**	**Data source within publication**
Plasmid *Spy*Cas9 to edit *Fah*[20]	2	Supplementary Figure 3
Plasmid ABE Base Editor to edit *Fah*[21]	6	Fig. 2
mRNA *Spy*Cas9 to edit *Fah*[22]	4	Fig. 3
AAV *Sau*Cas9 to edit *Otc*[23]	6	Fig. 3
RNP *Spy*Cas9 to edit *Pten*[24]	12	Fig. 3h
Gene correction via Cas9 and MMEJ to edit *Fah*[25]	4	Text (Page 121)
Plasmid *Nme*Cas9 to edit *Hpd*[26]	5	Fig. 2d
mRNA LNP *Sau*Cas9 to edit *Icam-2*[27]	4	Fig. 4
AAV N-int-BE3 to edit *Pah*[28]	4	Fig. 3e
**Total**	**47**	

Supplementary Table 9: Simbiology Project Files Utilized for GETEM.

**Gene Therapy Modality Being Modeled**	**Filename**
Plasmid *Spy*Cas9 to edit *Fah*	FAH_Editing_Project_v14_Yin_2014.sbproj
Plasmid ABE Base Editor to edit *Fah*	FAH_Editing_Project_v16_Song_2018.sbproj
mRNA *Spy*Cas9 to edit *Fah*	FAH_Editing_Project_v15_Yin_2016.sbproj
AAV *Sau*Cas9 to edit *Otc*	FAH_Editing_Project_v13_Yang_2016_AAV.sbproj
RNP *Spy*Cas9 to edit *Pten*	PTEN_Editing_Project_Wei_2020.sbproj
Gene correction via Cas9 and MMEJ to edit *Fah*	FAH_Editing_Project_Shin_2018.sbproj
Plasmid *Nme*Cas9 to edit *Hpd*	HPD_Editing_Project_Ibraheim_2018.sbproj
mRNA LNP *Sau*Cas9 to edit *Icam-2*	ICAM_2_Editing_Sago_2018.sbproj
AAV N-int-BE3 to edit *Pah*	PAH_Editing_Villiger_et_al_2018.sbproj
Pompe gene correction	Pompe_Model_v28.sbproj
Pompe gene correction + Cell Therapy	Pompe_Model_v30_CellDeath.sbproj

Supplementary Table 10: List of primers utilized in this study

Name	Primer F (5`-3`)	Primer R (5`-3`)
1441delT genomic	AGCTGCTCATTGACCTCCAG	CAATCCACATGCCGTCGAAG
2237G>A genomic	AATTCAGCCTCTTCCTGTGC	CATACGTTCCTCTTTCCGCC
Full length genomic	TGACAGGTTTCCCTCTTCCCAG	TTGATAACCTACACTGCGGGGG
1441delT qPCR/NGS	AGTGGGGCTTCCATGCAG	GGTTGGTGAAGTCGGGGAAG
2237G>A qPCR/NGS	CCAAGGACTCTAGCACCTGGAC	GGGAAGTAGCCAGTCACTTCGG
W746X NGS	TCCCATTCATCACCCGTATGC	AGGTCGTACCATGTGCCCAA
D645N R660H NGS	CTGAGGACCAGCCTGACTCT	CCACCCTACCAGACTGAGCA
2237-OT1	CCCTCCTCTGTGTGCCATTA	GTGCCATATTTTGGGGACCAC
2237-OT2	GGGGCATGGTCAGATGATGG	CACAGAAATTCCTGAGGCCAAC
2237-OT3	GGAGAGGCTGACCTTCATGG	TCGTGCTTTCCTGACCATCG
2237-OT4	CCTGGGGGAAAGGTAAAAGA	GCTGGAATGGTCTCGATCTC
2237-OT5	CACCCTGGAGTAGGCTTTCA	AGGACAGTGCCCTCACAATC
2237-OT6	ATGACCTCTGGGAATGCAGA	TCTGATGCTCCCTCAGTCCT
2237-OT7	CCCATTGCAACTGTGAACAA	TCTCCAACCATCAAGGAACA
2237-OT8	TCGCTCTGAAATGGGGATAG	CATATTTCCGGCACCATCTT
2237-OT9	CGTGTCTAGCCCCATCTCTT	CCTCCTCCCTGGTCCTAAAC
2237-OT10	GGGGAGCTTACCACCTTAGC	GCCTCTGTCTTCCAAATTGC
1441-OT1	AGTGTGCTTCCACTGTCGTT	GTGCGGGTAACCTTCTCCAT
1441-OT2	TTCCTCTGCTGCTGAGTTGG	GCCGATTAAAAGGCTGTCGC
1441-OT3	AGAGCCCTGGAGGTCATTGT	CTGTCTGGCCTCTGAATCGG
1441-OT4	ATTAGCCGGTGCCATGATAA	CGAGGAGCAACAGGGTAGAG
1441-OT5	GGGCAGTGTACCAGGTTAGG	GGTCCCCTAGGGTTAGCTTG

1441-OT6	CTGGACGACCTGAGCATTTT	AGAGTTCCACTTGGCCAGAA
1441-OT7	CATTCCACAGAGCCTGGTTT	ACTGCATAGGGATGGGACTG
1441-OT8	CACTTTCCCCAGCTCTTCAG	ATGGCACACCTGGTCCTAAC
1441-OT9	TCTCCACACGTGTTCCAATC	AAATATACACGGCCCACACC
1441-OT10	CTGTCCTCAGCCAACAGTGA	GTGTTCACAGCCAACACAGG

References for Supplementary Information

1. Sun, B. *et al.* Enhanced Response to Enzyme Replacement Therapy in Pompe Disease after the Induction of Immune Tolerance. *Am. J. Hum. Genet.* **81**, 1042–1049 (2007).
2. Raben, N. *et al.* Enzyme replacement therapy in the mouse model of Pompe disease. *Mol. Genet. Metab.* **80**, 159–169 (2003).
3. Lim, J.-A., Li, L. & Raben, N. Pompe disease: from pathophysiology to therapy and back again. *Front. Aging Neurosci.* **6**, 177 (2014).
4. Berrier, K. L. *et al.* CRIM-negative infantile Pompe disease: characterization of immune responses in patients treated with ERT monotherapy. *Genet. Med.* (2015) doi:10.1038/gim.2015.6.
5. Han, S. *et al.* Low-Dose Liver-Targeted Gene Therapy for Pompe Disease Enhances Therapeutic Efficacy of ERT via Immune Tolerance Induction. *Mol. Ther. - Methods Clin. Dev.* **4**, 126–136 (2017).
6. Martin-Touaux, E. *et al.* Muscle as a putative producer of acid α-glucosidase for glycogenosis type II gene therapy. *Hum. Mol. Genet.* **11**, 1637–1645 (2002).
7. Ding, E. y. *et al.* Long-Term Efficacy after [E1-, polymerase-] Adenovirus-Mediated Transfer of Human Acid-α-Glucosidase Gene into Glycogen Storage Disease Type II Knockout Mice. *Hum. Gene Ther.* **12**, 955–965 (2001).
8. Xu, F. *et al.* Glycogen storage in multiple muscles of old GSD-II mice can be rapidly cleared after a single intravenous injection with a modified adenoviral vector expressing hGAA. *J. Gene Med.* **7**, 171–178 (2005).
9. van der Wal, E., Bergsma, A. J., Pijnenburg, J. M., van der Ploeg, A. T. & Pijnappel, W. W. M. P. Antisense Oligonucleotides Promote Exon Inclusion and Correct the Common c.-32-13T>G GAA Splicing Variant in Pompe Disease. *Mol. Ther. - Nucleic Acids* **7**, 90–100 (2017).
10. van der Wal, E. *et al.* Large-Scale Expansion of Human iPSC-Derived Skeletal Muscle Cells for Disease Modeling and Cell-Based Therapeutic Strategies. *Stem Cell Rep.* doi:10.1016/j.stemcr.2018.04.002.

11. Carlson-Stevermer, J. *et al.* High-Content Analysis of CRISPR-Cas9 Gene-Edited Human Embryonic Stem Cells. *Stem Cell Rep.* **6**, 109–120 (2016).

12. Wang, B., Zhao, L., Fish, M., Logan, C. Y. & Nusse, R. Self-renewing diploid Axin2+ cells fuel homeostatic renewal of the liver. *Nature* **524**, 180–185 (2015).

13. Pryce, J. W. *et al.* Reference ranges for organ weights of infants at autopsy: results of >1,000 consecutive cases from a single centre. *BMC Clin. Pathol.* **14**, 18 (2014).

14. Bianconi, E. *et al.* An estimation of the number of cells in the human body. *Ann. Hum. Biol.* **40**, 463–471 (2013).

15. Chung, S. T., Chacko, S. K., Sunehag, A. L. & Haymond, M. W. Measurements of Gluconeogenesis and Glycogenolysis: A Methodological Review. *Diabetes* **64**, 3996–4010 (2015).

16. Usher, R., Shephard, M. & Lind, J. The Blood Volume of the Newborn Infant and Placental Transfusion. *Acta Paediatr.* **52**, 497–512 (1963).

17. Umapathysivam, K., Hopwood, J. J. & Meikle, P. J. Determination of Acid α-Glucosidase Activity in Blood Spots as a Diagnostic Test for Pompe Disease. *Clin. Chem.* **47**, 1378–1383 (2001).

18. Kishnani, P. *et al.* Duvoglustat HCl Increases Systemic and Tissue Exposure of Active Acid α-Glucosidase in Pompe Patients Co-administered with Alglucosidase α. *Mol. Ther.* **25**, 1199–1208 (2017).

19. Albayrak, C. *et al.* Digital Quantification of Proteins and mRNA in Single Mammalian Cells. *Mol. Cell* **61**, 914–924 (2016).

20. Yin, H. *et al.* Genome editing with Cas9 in adult mice corrects a disease mutation and phenotype. *Nat. Biotechnol.* **32**, 551–553 (2014).

21. Song, C.-Q. *et al.* Adenine base editing in an adult mouse model of tyrosinaemia. *Nat. Biomed. Eng.* 1 (2019) doi:10.1038/s41551-019-0357-8.

22. Yin, H. *et al.* Therapeutic genome editing by combined viral and non-viral delivery of CRISPR system components in vivo. *Nat. Biotechnol.* **advance on**, (2016).

23. Yang, Y. *et al.* A dual AAV system enables the Cas9-mediated correction of a metabolic liver disease in newborn mice. *Nat. Biotechnol.* **34**, 334–338 (2016).

24. Wei, T., Cheng, Q., Min, Y.-L., Olson, E. N. & Siegwart, D. J. Systemic nanoparticle delivery of CRISPR-Cas9 ribonucleoproteins for effective tissue specific genome editing. *Nat. Commun.* **11**, 3232 (2020).

25. Shin, J. H., Jung, S., Ramakrishna, S., Kim, H. H. & Lee, J. In vivo gene correction with targeted sequence substitution through microhomology-mediated end joining. *Biochem. Biophys. Res. Commun.* **502**, 116–122 (2018).

26. Ibraheim, R. *et al.* All-in-one adeno-associated virus delivery and genome editing by Neisseria meningitidis Cas9 in vivo. *Genome Biol.* **19**, 137 (2018).

27. Sago, C. D. *et al.* High-throughput in vivo screen of functional mRNA delivery identifies nanoparticles for endothelial cell gene editing. *Proc. Natl. Acad. Sci.* **115**, E9944–E9952 (2018).

28. Villiger, L. *et al.* Treatment of a metabolic liver disease by in vivo genome base editing in adult mice. *Nat. Med.* **24**, 1519–1525 (2018).

29. Mitzelfelt, K. A. *et al.* Efficient Precision Genome Editing in iPSCs via Genetic Co-targeting with Selection. *Stem Cell Rep.* **8**, 491–499 (2017).

30. Mollova, M. *et al.* Cardiomyocyte proliferation contributes to heart growth in young humans. *Proc. Natl. Acad. Sci. U. S. A.* **110**, 1446–1451 (2013).

31. Råsten-Almqvist, P., Eksborg, S. & Rajs, J. Heart weight in infants-a comparison between Sudden Infant Death Syndrome and other causes of death. *Acta Paediatr.* **89**, 1062–1067 (2007).

32. Raben, N. *et al.* Replacing acid α-glucosidase in Pompe disease: recombinant and transgenic enzymes are equipotent, but neither completely clears glycogen from type II muscle fibers. *Mol. Ther.* **11**, 48–56 (2005).

33. Haus, J. M., Carrithers, J. A., Carroll, C. C., Tesch, P. A. & Trappe, T. A. Contractile and connective tissue protein content of human skeletal muscle: effects of 35 and 90 days of simulated microgravity

and exercise countermeasures. *Am. J. Physiol.-Regul. Integr. Comp. Physiol.* **293**, R1722–R1727 (2007).

34. Hribal, M. L., Oriente, F. & Accili, D. Mouse models of insulin resistance. *Am. J. Physiol.-Endocrinol. Metab.* **282**, E977–E981 (2002).
35. Falk, D. J. *et al.* Comparative impact of AAV and enzyme replacement therapy on respiratory and cardiac function in adult Pompe mice. *Mol. Ther. Methods Clin. Dev.* **2**, 15007 (2015).
36. Partridge, T. A. Cells that participate in regeneration of skeletal muscle. *Gene Ther.* **9**, 752–753 (2002).
37. Kishnani, P. S., Sun, B. & Koeberl, D. D. Gene therapy for glycogen storage diseases. *Hum. Mol. Genet.* **28**, R31–R41 (2019).
38. Blanco, E., Shen, H. & Ferrari, M. Principles of nanoparticle design for overcoming biological barriers to drug delivery. *Nat. Biotechnol.* **33**, 941–951 (2015).
39. Kroon, E. *et al.* Pancreatic endoderm derived from human embryonic stem cells generates glucose-responsive insulin-secreting cells in vivo. *Nat. Biotechnol.* **26**, 443–452 (2008).
40. Bierwolf, J. *et al.* Primary Human Hepatocytes Repopulate Livers of Mice After In Vitro Culturing and Lentiviral-Mediated Gene Transfer. *Tissue Eng. Part A* **22**, 742–753 (2016).
41. Growth Charts - Clinical Growth Charts. https://www.cdc.gov/growthcharts/clinical_charts.htm.
42. Chien, Y.-H. *et al.* Long-Term Prognosis of Patients with Infantile-Onset Pompe Disease Diagnosed by Newborn Screening and Treated since Birth. *J. Pediatr.* **166**, 985-991.e2 (2015).

708
