# Supplementary figures and images for: Design of efficacious somatic cell genome editing strategies for recessive and polygenic diseases
Source: Nat Commun. 2020 Dec 8;11:6277. doi: 10.1038/s41467-020-20065-8 (PMC7722885; doi:10.1038/s41467-020-20065-8)

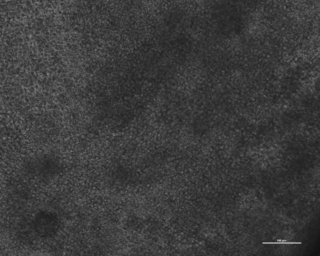

Supplement: Supplementary file 4 — Supplementary Movie 1 [file 41467_2020_20065_MOESM4_ESM.gif]

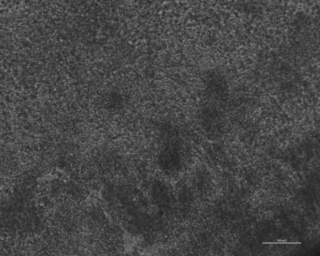

Supplement: Supplementary file 5 — Supplementary Movie 2 [file 41467_2020_20065_MOESM5_ESM.gif]

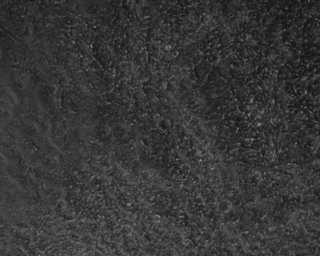

Supplement: Supplementary file 6 — Supplementary Movie 3 [file 41467_2020_20065_MOESM6_ESM.gif]

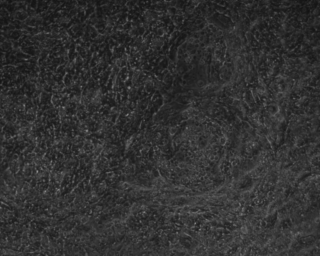

Supplement: Supplementary file 7 — Supplementary Movie 4 [file 41467_2020_20065_MOESM7_ESM.gif]
