# Supplementary material for: Design of efficacious somatic cell genome editing strategies for recessive and polygenic diseases
Source: Nat Commun. 2020 Dec 8;11:6277. doi: 10.1038/s41467-020-20065-8 (PMC7722885; doi:10.1038/s41467-020-20065-8)
Supplement: Supplementary file 8 — Description of Additional Supplementary Files [file 41467_2020_20065_MOESM8_ESM.pdf]

**Title:** Supplementary Movie 1

**Description:** Graphics Interchange Format video demonstrating the spontaneous beating of unedited Pompe disease iPSC derived cardiomyocytes.

**Title:** Supplementary Movie 2

**Description:** Graphics Interchange Format video demonstrating the spontaneous beating of 746 Correct iPSC derived cardiomyocytes

**Title:** Supplementary Movie 3

**Description:** Graphics Interchange Format video demonstrating the spontaneous beating of dT Correct iPSC derived cardiomyocytes

**Title:** Supplementary Movie 4

**Description:** Graphics Interchange Format video demonstrating the spontaneous beating of DoubleCorrect.c1 iPSC derived cardiomyocytes
